# Co-Inactivation of GlnR and CodY Regulators Impacts Pneumococcal Cell Wall Physiology

**DOI:** 10.1371/journal.pone.0123702

**Published:** 2015-04-22

**Authors:** Calum Johnston, Hester J. Bootsma, Christine Aldridge, Sylvie Manuse, Nicolas Gisch, Dominik Schwudke, Peter W. M. Hermans, Christophe Grangeasse, Patrice Polard, Waldemar Vollmer, Jean-Pierre Claverys

**Affiliations:** 1 Centre National de la Recherche Scientifique, LMGM-UMR5100, F-31000 Toulouse, France; 2 Université de Toulouse, UPS, Laboratoire de Microbiologie et Génétique Moléculaires, F-31000 Toulouse, France; 3 Laboratory of Pediatric Infectious Diseases, Radboud University Medical Centre, 6500 HB Nijmegen, The Netherlands; 4 Centre for Bacterial Cell Biology, Institute for Cell and Molecular Biosciences, Newcastle University, Richardson Road, Newcastle upon Tyne NE2 4AX, United Kingdom; 5 Bases Moléculaires et Structurales des Systèmes Infectieux, UMR 5086, Université Lyon 1, CNRS, IBCP, F-69007 Lyon, France; 6 Research Center Borstel, Leibniz-Center for Medicine and Biosciences, 23845 Borstel, Germany; Centers for Disease Control & Prevention, UNITED STATES

## Abstract

CodY, a nutritional regulator highly conserved in low G+C Gram-positive bacteria, is essential in *Streptococcus pneumoniae* (the pneumococcus). A published *codY* mutant possessed suppressing mutations inactivating the *fatC* and *amiC* genes, respectively belonging to iron (Fat/Fec) and oligopeptide (Ami) ABC permease operons, which are directly repressed by CodY. Here we analyzed two additional published *codY* mutants to further explore the essentiality of CodY. We show that one, in which the regulator of glutamine/glutamate metabolism *glnR* had been inactivated by design, had only a suppressor in *fecE* (a gene in the *fat/fec *operon), while the other possessed both *fecE* and *amiC* mutations. Independent isolation of three different *fat/fec* suppressors thus establishes that reduction of iron import is crucial for survival without CodY. We refer to these as primary suppressors, while inactivation of *ami*, which is not essential for survival of *codY* mutants and acquired after initial *fat/fec* inactivation, can be regarded as a secondary suppressor. The availability of *codY* ^-^ *ami* ^+^ cells allowed us to establish that CodY activates competence for genetic transformation indirectly, presumably by repressing *ami* which is known to antagonize competence. The *glnR codY fecE* mutant was then found to be only partially viable on solid medium and hypersensitive to peptidoglycan (PG) targeting agents such as the antibiotic cefotaxime and the muramidase lysozyme. While analysis of PG and teichoic acid composition uncovered no alteration in the *glnR codY fecE* mutant compared to wildtype, electron microscopy revealed altered ultrastructure of the cell wall in the mutant, establishing that co-inactivation of GlnR and CodY regulators impacts pneumococcal cell wall physiology. In light of rising levels of resistance to PG-targeting antibiotics of natural pneumococcal isolates, GlnR and CodY constitute potential alternative therapeutic targets to combat this debilitating pathogen, as co-inactivation of these regulators renders pneumococci sensitive to iron and PG-targeting agents.

## Introduction

The global nutritional regulator CodY is highly conserved in low G+C Gram-positive bacteria [1], and regulates up to 200 genes in *Bacillus subtilis* [2]. The *B*. *subtilis* CodY regulon concerns not only metabolic pathways, but also cellular processes such as sporulation, motility and competence for genetic transformation [1,3,4]. Most of these genes are directly repressed by CodY during exponential growth and induced upon nutrient starvation. In other species, CodY has also been shown to regulate a number of major virulence genes (for reviews, see references [1,3]) by directly binding DNA and repressing the target genes. CodY is activated by branched chain amino acids [5] but also by GTP in certain species, such as *B*. *subtilis* [6].

Transcriptome analysis of a *codY* mutant in the human pathogen *Streptococcus pneumoniae* showed that CodY mainly regulated amino acid metabolism, biosynthesis and uptake [7]. However, it was recently demonstrated that the *codY* mutant used in this study had accumulated suppressing mutations allowing tolerance of *codY* inactivation (collectively called *socY* for suppressor of *c* *od* *Y*), and that the *codY* gene could not be readily inactivated by insertion of an antibiotic cassette [8]. A first suppressing mutation was identified in the *fatC* gene by whole-genome sequencing of the *codY* mutant [8]. This gene belongs to the *fatD*–*fatC*–*fecE*–*fatB* operon; this operon (also called *piuBCDA* or *pit1*) [9], which is directly repressed by CodY [7], encodes the major ferric iron ABC permease of *S*. *pneumoniae* [10], with FatB also shown to bind heme [11]. While the *fatC* mutation was present in the entire *codY* ^-^ population, a second suppressing, variable mutation was found in the *amiC* gene [8], encoding a subunit of the Ami oligopeptide ABC permease [12]. It was concluded that the three different *amiC* mutations identified in the *codY* ^-^ population arose subsequently to *fatC* in an otherwise *codY fatC* mutant lineage, presumably providing a selective advantage over *ami* ^+^ cells. Based on these data, CodY was suggested to be an essential protein in *S*. *pneumoniae* primarily because repression of the *fat*/*fec* operon by CodY was required to avoid uncontrolled iron import resulting in toxicity [8].

Two further pneumococcal *codY* mutant strains have been published [13,14], including one in which *glnR*, encoding a regulator of glutamine/glutamate (Glu/Gln) metabolism, was also inactivated by design [13]. Because CodY was previously concluded to be an essential protein in *S*. *pneumoniae* [8], we analyzed these mutants to establish whether new suppressing mutations allowed tolerance of *codY* inactivation in these strains. Here we show that both strains contain mutations truncating the *fecE* gene, encoding another subunit of the Fat/Fec permease. The independent isolation of three different mutations in the *fat/fec* operon crucial for tolerance of *codY* inactivation demonstrates that *codY* essentiality results from the deregulation of iron import when CodY is absent and unable to repress *fat/fec*. Furthermore, we provide evidence that the published *glnR codY* mutant, while possessing no other suppressor than the *fecE* mutation, is only partially viable on solid medium. We establish that this reduced/poor viability is linked to the co-inactivation of *codY* and *glnR*. This mutant was also found to be hypersensitive to the peptidoglycan (PG) targeting antibiotic cefotaxime, as well as to the PG-targeting muramidase enzyme lysozyme, to which the pneumococcus is normally naturally resistant [15]. Although biochemical analysis revealed normal PG composition in the mutant, electron microscopy analysis showed mutant cells to have thicker, irregular and yet less dense cell wall leading us to conclude that co-inactivation of GlnR and CodY regulators impacts pneumococcal cell wall physiology.

## Results

### Genetic dissection of the *glnR codY* double mutant

**Fig 1:**
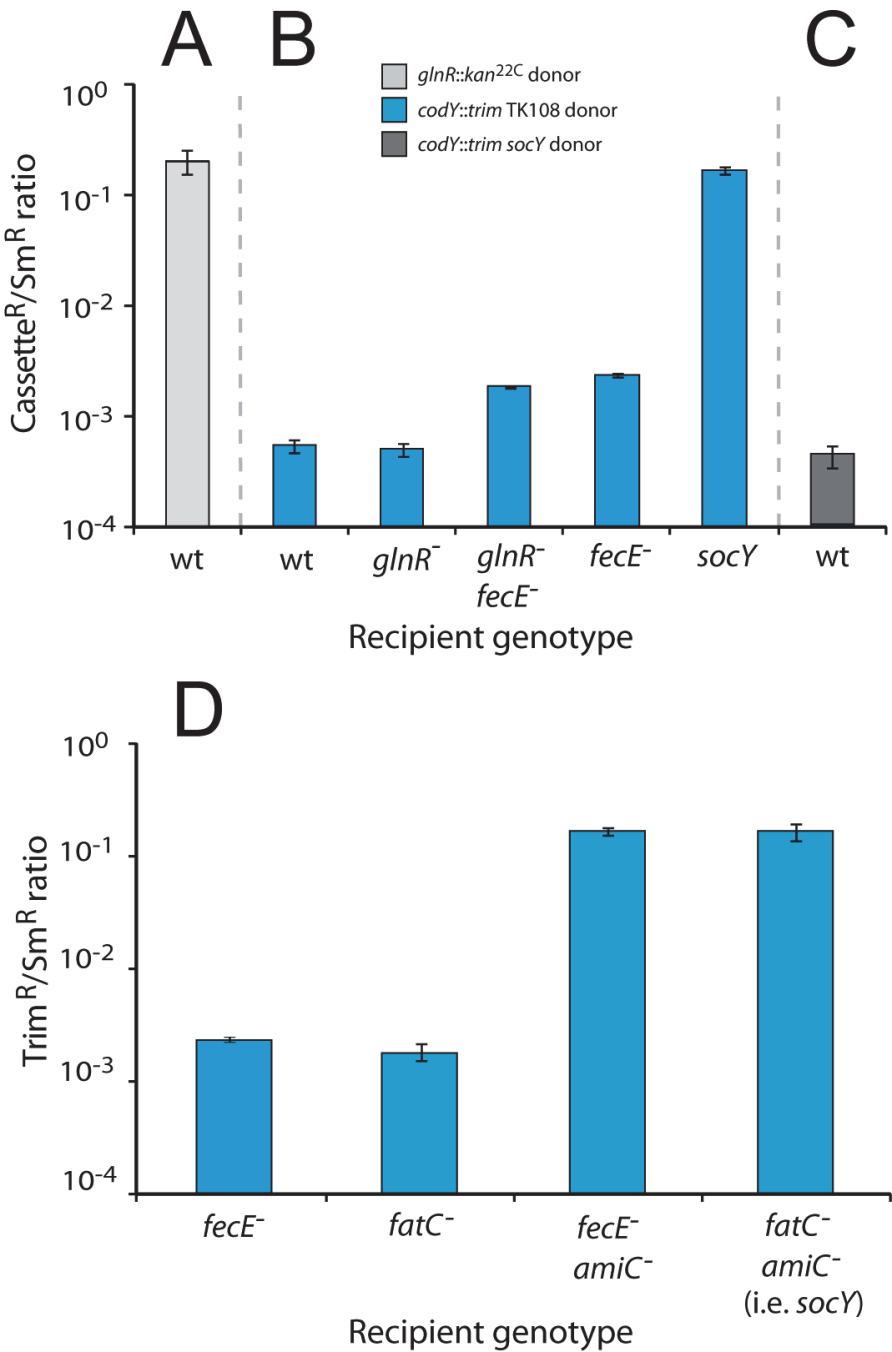
Genetic dissection of *codY* mutants. (A) Transformation efficiency of the *glnR*::*kan* ^22C^ cassette into wildtype (wt) recipient. Transformation efficiencies calculated from triplicate repeats. Donor strain, R3154; wt recipient strain, TD198. (B) Transformation efficiency of the *codY*::*trim* cassette present in the *glnR codY* (*fecE*) mutant into wt and mutant recipient strains. Transformation efficiencies calculated as in panel A. Donor strain, TD196 (i.e. TK108 but *rpsL41*). Recipient strains, wt, TD198; *glnR* ^-^, TD195; *glnR* ^-^ *fecE* ^-^, TD227; *fecE* ^-^, TD230; *socY*, TD142. (C) Transformation efficiency of the *codY*::*trim* cassette from the suppressed *codY*::*trim*, *socY* strain into a wt recipient. Transformation efficiencies calculated as in panel A. Donor strain, TD80; wt recipient as in panel A. (D) Inactivation of *fatC* and *fecE* has the same suppressive effect on *codY* inactivation. Transformation efficiency of the *codY*::*trim* cassette present in the TK108 mutant into wt and mutant recipient strains. Transformation efficiencies calculated as in panel A. Donor strain, TD196. Recipient strains, *fecE* ^-^, TD230; *fatC* ^-^, TD141; *fecE* ^-^ *amiC* ^-^, TD228; *socY*, TD142.

We began our study by investigating whether inactivation of *glnR* could be responsible for suppressing the essentiality of *codY* in strain TK108, the published D39 *glnR codY*::*trim* double mutant [13]. We initially determined that a *glnR*::*kan* ^22C^ cassette, conferring kanamycin resistance (Kan^R^), transformed with normal efficiency into a wildtype recipient strain (Fig 1A). Throughout this study, transformation efficiencies of mutations were always compared to the same reference marker, the *rpsL41* point mutation, conferring streptomycin resistance (Sm^R^) [16] and carried on the same donor DNA as the mutation under investigation. An efficiency of 0.25 for the Kan^R^ cassette relative to Sm^R^ (Fig 1A) is in the range expected for transformation of a heterologous insert compared to a point mutation [17], showing that *glnR* could readily be inactivated without additional suppressing mutations.

To establish whether the inactivation of *glnR* could suppress *codY* essentiality, we transformed a *glnR* mutant with genomic DNA from TD196 (i.e. TK108 but *rpsL41*; S1 Table). The transformation efficiency of *codY*::*trim* (conferring trimethoprim resistance, Trim^R^) was over 350-fold lower than normal efficiency and was comparable to that observed in a wildtype recipient (Fig 1B). This result established that mutation of *glnR* does not allow cells to tolerate *codY* inactivation.

Interestingly, the wildtype recipient was transformed with similar efficiency using as donor TD196 or the previously characterized *codY*::*trim socY* strain (Fig 1C). Since in the latter case survival of *codY* ^-^ transformants required the simultaneous co-transfer of two independent suppressors (the *amiC* and *fatC* mutations to be referred to as *socY* hereafter) [8], explaining the very low frequency of Trim^R^ clones recovered, these results suggested that yet uncharacterized suppressing mutations could also be present in the *glnR codY* mutant to allow tolerance of *codY* inactivation.

### A *fecE* mutation suppresses inviability of *codY* mutants

Sequence analysis of targeted PCRs showed that the *glnR codY* mutant possessed wildtype *fatC* and *amiC* genes, suggesting the presence of alternative suppressor(s) than the previously detailed *socY* mutations. Whole-genome sequencing of TK108 was then used (Materials and Methods), identifying only 6 single nucleotide polymorphisms (SNPs) compared to D39 [18], and no mutation in the entire *ami* operon. Localized PCR and sequencing of TK102, the *glnR* ^-^ parent of TK108, showed that 5 of the 6 SNPs were already present prior to *codY*::*trim* transformation and thus unlikely to be involved in suppression of *codY* essentiality. Interestingly, the only remaining mutation was a C→T mutation introducing a stop codon in the *fecE* gene (referred to as *fecE* ^-^ hereafter), resulting in the truncation of the FecE protein after 13 of 250 amino acids. This gene is part of the *fat/fec* iron permease operon, as is *fatC*. It seemed therefore likely that the *fecE* mutation was responsible for tolerance of *codY* inactivation.

To determine whether *fecE* ^-^ suppressed *codY* essentiality, the point mutation was introduced into the *glnR* ^-^ parent without selection (Materials and Methods) to create a *glnR fecE* mutant (TD227). The *codY*::*trim* cassette was transformed into both *fecE* ^-^ and *glnR* ^-^ *fecE* ^-^ recipient strains, and results showed that this cassette transferred with ∼10-fold higher efficiency into both strains than into the *glnR* ^-^ parent or the wild type (Fig 1B). However, this efficiency remained lower than into the fully suppressed *socY* recipient strain, showing that *fecE* ^-^ only partially suppressed *codY*::*trim* lethality. To confirm the equivalence of *fecE* and *fatC* mutations as suppressors of *codY* ^-^ inviability, the *fecE* ^-^ mutation was inserted into a strain mutant for *amiC*, thus recreating a *socY*-like combination, and the transformation of *codY*::*trim* repeated. Unlike both *fatC* and *fecE* single mutants, the *fecE amiC* and *fatC amiC* double mutants readily accepted the *codY*::*trim* cassette, showing that both mutations of the *fat/fec* operon are equally suppressive of *codY*::*trim* lethality, resulting in full suppression in conjunction with *amiC* mutation (Fig 1D).

Interestingly, a third pneumococcal *codY* mutant was recently published [14]. These authors showed that this *codY* mutant harbored a mutation in the *amiC* gene [14] but possessed a wildtype *fatC* gene (Sven Hammerschmidt, personal communication). However, we discovered by sequencing that a deletion of base 409 (G) resulted in truncation of the *fecE* gene in this *codY* mutant. Thus, all three independently constructed *codY* mutants of *S*. *pneumoniae* possess an inactive *fat/fec* encoded iron permease, providing strong evidence that this inactivation is necessary to allow pneumococci to tolerate *codY* inactivation. Since it is impossible to create a pneumococcal strain with only *glnR* and *codY* inactivated, we will hereafter refer to the mutant under study as *glnR* ^-^ *codY* ^-^ (*fecE* ^-^) to highlight the presence of an accompanying necessary suppressing mutation in *fecE*.

### The *glnR codY* (*fecE*) mutant displays a severe colony-forming defect

**Fig 2:**
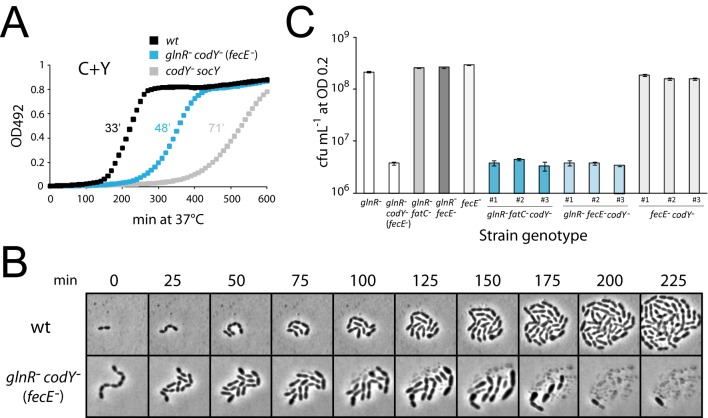
The *glnR codY fecE* triple mutant is only partially viable on solid medium. (A) Growth curves of strains in C+Y medium. After initial growth to identical cellular densities (OD 0.2) in C+Y medium, cells were diluted 1/100 in 300 μL final volume of C+Y medium in microtitre plate, and OD_492_ readings taken every 10 min for 600 min. Strain identities, wt, D39; *glnR* ^-^ *codY* ^-^ (*fecE* ^-^), TK108; *codY* ^-^ *socY*, TD75. (B) Time-lapse microscopy of wildtype and *codY* mutant cells growing in CAT-agarose. Strain identities as in panel A. (C) Loss of viability is maintained in newly generated *glnR fecE codY* triple mutants. Viable counts performed on newly created *codY*::*trim* transformants with varying parental genotypes. Colony-forming units per ml (cfu mL^-1^) counted after growth to OD_550_ 0.2 in C+Y and plating on CAT agar + 5% horse blood. From left to right, columns 1 and 2 represent viable (TD195) and non-viable (TK108) controls; columns 3–5 represent parent strains (TD212 black, TD227 dark grey and TD230 light grey respectively); three Trim^R^ transformant clones (appropriately coloured panels numbered 1–3) from each parent transformed by *codY*::*trim* PCR fragment. Average cfu mL^-1^ calculated from triplicate repeats.

Comparison of growth of *glnR* ^-^ *codY* ^-^ (*fecE* ^-^), *codY* ^-^ *socY* and wildtype strains in liquid media (C+Y, CAT, TSB and THY) showed that *codY* mutants grew slower than wildtype cells in all instances (Fig 2A and S1 Fig). However, the *codY socY* mutant generally grew even slower and/or displayed a longer lag than its *glnR codY* (*fecE*) counterpart (particularly in C+Y medium, Fig 2A). This supported our conclusion that *glnR* ^-^ *codY* ^-^ (*fecE* ^-^) cells were *ami* ^+^ as inferred from whole-genome sequencing data, since *ami* ^-^ cells have been repeatedly observed over years in our laboratory to grow significantly slower than wildtype cells.

We then analyzed *glnR codY* (*fecE*) mutant cells by fixing them on microscope slides using polylysin (Materials and Methods), and exploring their morphology (S1 Text and S2 and S3 Figs). Mutant cells appeared to form short chains, with an average number of cells per chain of 4.6 compared to 1.7 for the wild type (S2G Fig), where one chain should give rise to one cfu after plating. Notably, we observed a ~3-fold reduction in individual cell numbers (counted irrespective of whether these were part of chains or not) compared to wild type for aliquots from cultures of the same initial optical density, suggesting that a significant proportion (>66%) of the *glnR codY* (*fecE*) mutant cells lysed in these conditions (S2F Fig). To account for such a deficit in cell numbers, which is unlikely to come from a loss of viability in liquid culture since cells were grown to the same OD and we do not see a substantial difference in doubling time in liquid culture (Fig 2A), we suggest that fixing the cells on microscope slides using polylysin may have caused lysis of a fraction of the population. In contrast to *glnR codY* (*fecE*) mutant cells, the *codY* ^-^ *socY* mutant while also forming short chains (average number of cells per cfu of 5.7; Fig 2G) showed only a modest deficit (∼25%) in individual cell numbers compared to wild type (as well as to a *glnR fecE* mutant and a *codY* ^+^ complemented *glnR codY* (*fecE*) mutant; S3E Fig), suggesting more limited cell lysis (S2F Fig). On the other hand, a *glnR fecE* mutant displayed characteristics similar to wildtype (S3 Fig).

Comparing viable counts of the *glnR codY* (*fecE*) mutant with wild type and the *codY socY* mutant then revealed that the former displayed a ~20-fold loss of colony-forming units (cfu) compared to wild type when plated on CAT-agar (a deficit also observed in THY-agar, and using either Select Agar from Invitrogen or Ultrapure Agarose from Sigma) (data not shown). Viable *glnR* ^-^ *codY* ^-^ (*fecE* ^-^) clones picked and regrown in liquid medium displayed the same loss of viability upon plating, indicating that these clones had not accumulated suppressing mutations but represented random low frequency survivors. In contrast, the *codY* ^-^ *socY* strain was fully viable, establishing that mortality is specific to the *glnR codY* (*fecE*) mutant. *glnR* ^-^ *codY* ^-^ (*fecE* ^-^) colonies were nevertheless similar in size to wild type, in contrast to *codY* ^-^ *socY*, where *ami* inactivation results in very small colonies, further confirming that the former were *ami*+. Altogether, these observations suggested that the loss of viability was unrelated to overall growth rate but could be associated with plating on solid medium.

To further document the loss of viability, time-lapse microscopy was used to visualize the morphology and growth of *glnR codY* (*fecE*) mutant cells on CAT-agarose (Materials and Methods). Firstly, the rather low cell density of these cultures compared to control culture revealed that the majority of cells lysed shortly (i.e. within less than 1 hour) after transition from liquid to solid medium, leaving only cell debris. Secondly, cells that did not lyse immediately displayed aberrant cell morphology, generally being significantly larger than wildtype cells, and started lysing after 75–100 min growth on solid medium (Fig 2B). Clearly some of these cells must survive, as colonies are formed on solid medium, but these results confirm that the majority of cells are rapidly lost.

To confirm that the colony-forming defect observed was really due to the co-inactivation of *codY* and *glnR* (and accompanying necessary suppressor mutation in *fecE* or *fatC*), we recreated strains by transforming a *codY*::*trim* PCR fragment into *glnR fatC*, *glnR fecE* and *fecE* mutants. Three Trim^R^ transformants were recovered from each transformation, and grown in identical conditions to determine viable counts. Results showed that unlike the generated *fecE codY* double mutants, the resulting *glnR fecE codY* and *glnR fatC codY* triple mutants all displayed a loss of viability upon plating (Fig 2C). These observations established that it is the simultaneous inactivation of *glnR* and *codY* which results in lysis and associated loss of viability in solid medium.

### Restoration of normal colony-forming ability of the *glnR codY* (*fecE*) mutant

**Fig 3:**
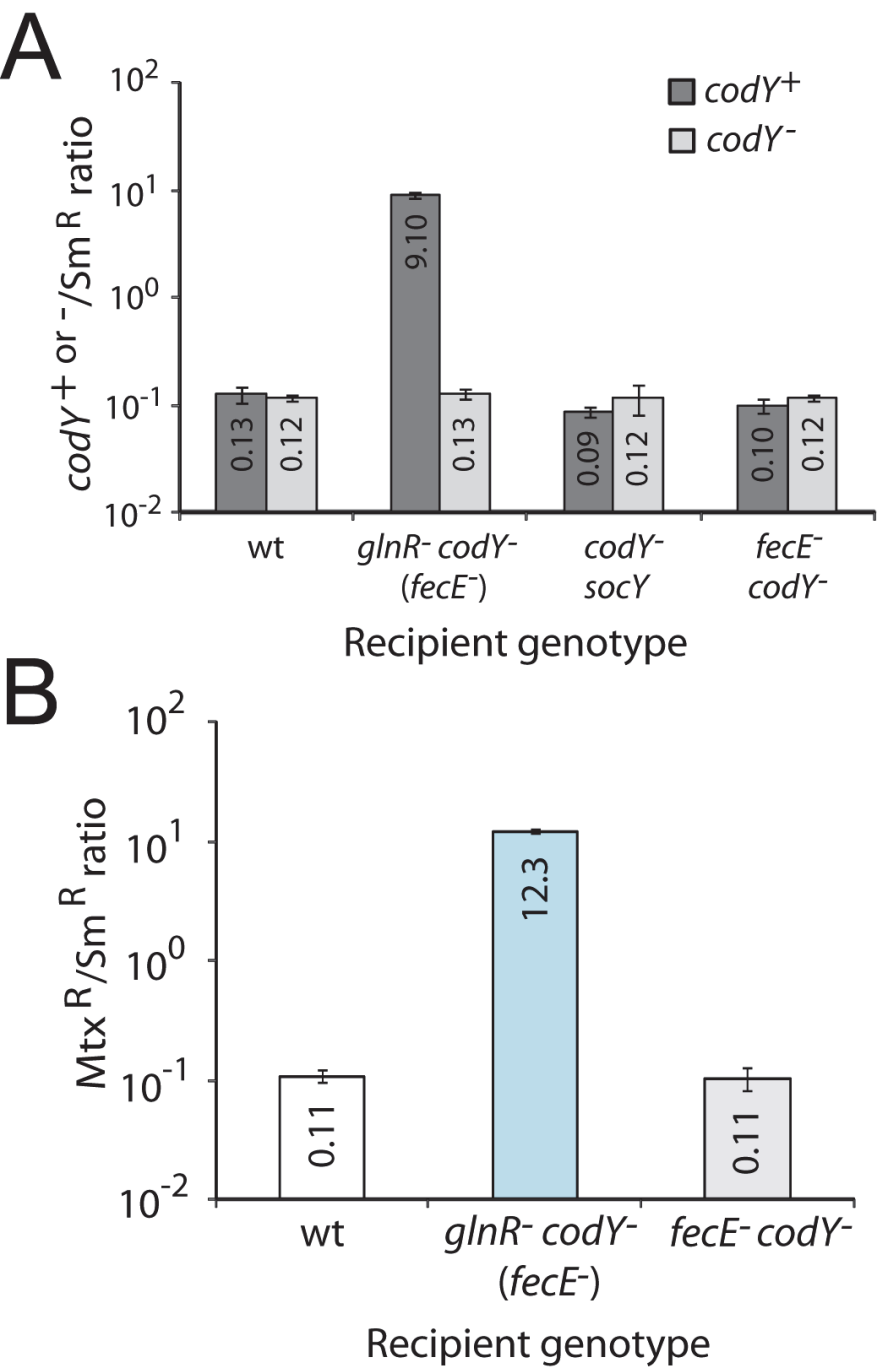
Restoration of normal colony-forming ability of the *glnR codY* (*fecE*) mutant. (A) Restoration by reintroduction of an ectopic, wildtype copy of *codY*. Ratios of Kan^R^ transformants obtained from transformation with either CEP_M_-*codY* ^*+*^ (dark grey bars, R2437 donor; indicated as *codY* ^+^) or CEP_M_-*codY*::*spc* ^3A^ (light grey bars, R3701; indicated as *codY* ^-^). Transformation efficiency of CEP_M_-*kan* cassettes normalized as in Fig 1A. Recipient strain identities, wt, D39; *glnR* ^-^ *codY* ^-^ (*fecE* ^-^), TK108; *codY* ^-^ *socY*, TD75. *fecE* ^-^ *codY* ^-^, TD268. Transformation efficiencies calculated from triplicate repeats. Recipient strains in this panel were *hexA* ^*+*^. (B) Inactivation of *ami* restores the viability of *glnR codY* (*fecE*) mutant cells. The *amiC9* mutation which inactivates the *amiC* gene renders the Ami permease inactive and confers resistance to methotrexate (Mtx^R^). Ratios of Mtx^R^ transformants normalized as in Fig 1A with transformation efficiencies calculated from triplicate repeats. Recipient strain identities, wt, D39; *glnR* ^-^ *codY* ^-^ (*fecE* ^-^), TK108. Recipient strains in this panel were *hexA* ^*+*^.

We then checked whether reintroduction of an ectopic, wildtype copy of *codY* into the *glnR codY* (*fecE*) mutant restored full viability. This experiment involved transformation with DNA from two strains in parallel, one possessing a CEP_M_ *-codY* ^*+*^ cassette introducing wildtype *codY* at an ectopic chromosomal location [19] and the other possessing the same cassette but with *codY* inactivated by insertion of a *mariner* minitransposon (CEP_M_ *-codY*::*spc* ^*3*A^). Transformation efficiency of CEP_M_-*codY* ^*+*^ was ~100-fold higher than CEP_M_ *-codY*::*spc*, establishing that introduction of ectopic *codY* ^+^ restored normal viability on solid medium (Fig 3A). The abnormally high *codY* ^+^/Sm^R^ ratio observed (almost 90-fold excess *codY* ^+^ cassette transformants over the expected number) providing strong support for this conclusion is fully explained by the fact that the Sm^R^ but not the *codY* ^+^ transformants display the *glnR* ^-^ *codY* ^-^ (*fecE* ^-^) specific colony-forming defect. A large proportion of Sm^R^ transformants are thus lost, skewing the resulting *codY* ^+^/Sm^R^ ratio.

Secondly, since *codY* can be readily inactivated in a *fecE amiC* mutant (Fig 1D), we hypothesized that inactivation of *ami* would rescue *glnR codY* (*fecE*) mutants from plating mortality. To check this, we compared the efficiency of transformation of *amiC9*, a point mutation inactivating the Ami permease, into wildtype and *glnR* ^-^ *codY* ^-^ (*fecE* ^-^) recipients. Whilst *amiC9* transformed into wild type with expected efficiency compared to Sm^R^, a ~110-fold excess in *amiC9* transformants over the expectation was observed in the triple mutant (Fig 3B), establishing that inactivation of *ami* restored colony-forming ability of *glnR codY* ^-^ (*fecE* ^-^) cells to the same extent as a second, ectopic copy of *codY*. In parallel, introduction of ectopic *codY* ^+^ and of *amiC9* into *fecE* ^-^ *codY* ^-^ cells occurred with efficiencies similar to wild type (Fig 3) providing further support to the conclusion that the mortality observed is linked to the co-inactivation of *codY* and *glnR*.

### CodY and competence for genetic transformation

The finding that the *fecE codY* double mutant was fully viable (Fig 2C) offered us the opportunity to reinvestigate the effect of *codY* inactivation on pneumococcal competence for genetic transformation [4,20], in the presence of a functional *ami* operon. CodY actively represses competence in *B*. *subtilis* [4,21], but a previous study in an *S*. *pneumoniae codY* mutant did not provide a clear answer as to whether CodY played a regulatory role on pneumococcal competence [8]. This is because at the time, the only characterized *codY* mutant was a *codY fatC amiC* mutant and *ami* mutations strongly upregulate competence [22], masking any potential role of CodY.

Competence profiles of *codY fecE* mutant cells were therefore analyzed throughout incubation at 37°C in C+Y medium with initial pH values between 6.6 and 7.6, since spontaneous competence induction is known to be strongly dependent on the initial pH [8,23] (Materials and Methods). The *codY fecE* mutant displayed a competence-down phenotype compared with wildtype and *fecE* mutant strains, as illustrated by the failure to develop competence at pH 6.9 and the strongly reduced competence at pH 7.1 (S4A—S4C Fig), suggesting that CodY may in fact activate pneumococcal competence.

Since CodY represses *ami* [7], and *ami* itself antagonizes spontaneous competence development [22], ‘activation’ of competence by CodY could rely primarily on repression of the *ami* operon. We therefore mutated the CodY binding site (*CYbs*) in the *ami* promoter, creating the *ami* ^*CYbs0*^ mutation to abolish repression of *ami* despite the presence of CodY (Materials and Methods). Monitoring competence of *ami* ^*CYbs0*^ and *fecE ami* ^*CYbs0*^ mutants revealed that *ami* derepression by the *ami* ^*CYbs0*^ mutation decreased competence in a similar manner to the inactivation of *codY* (S4D and S4E Fig). These results show that unlike in *B*. *subtilis*, CodY activates competence in *S*. *pneumoniae* but this activation is probably indirect, resulting from CodY-mediated repression of *ami*.

### Sensitivity of *glnR codY* (*fecE*) mutant cells to lytic enzymes and cefotaxime

We then wished to establish whether the rapid lysis of *glnR* ^-^ *codY* ^-^ (*fecE* ^-^) cells in solid medium was dependent on the major pneumococcal autolysin LytA. LytA is an amidase enzyme which cleaves the stem peptide of PG, separating it from the glycan strands at its base thereby disrupting the integrity of the cell wall and causing lysis [24,25]. A *lytA*::*cat* cassette [26] was transformed into the *glnR codY* (*fecE*) mutant and wild type, comparing its efficiency of transformation to Sm^R^. A ratio of ~0.1 was observed for both recipient strains (data not shown), indicating that in contrast to *ami* inactivation (Fig 3B), inactivation of *lytA* did not restore colony-forming ability of *glnR codY* ^-^ (*fecE* ^-^) cells. Moreover, three *glnR codY* (*fecE*) *lytA* transformants picked at random still displayed reduced colony-forming ability (data not shown). Both results established that LytA is neither responsible nor required for the lysis and resulting loss of viability of the triple mutant in solid medium.

**Fig 4:**
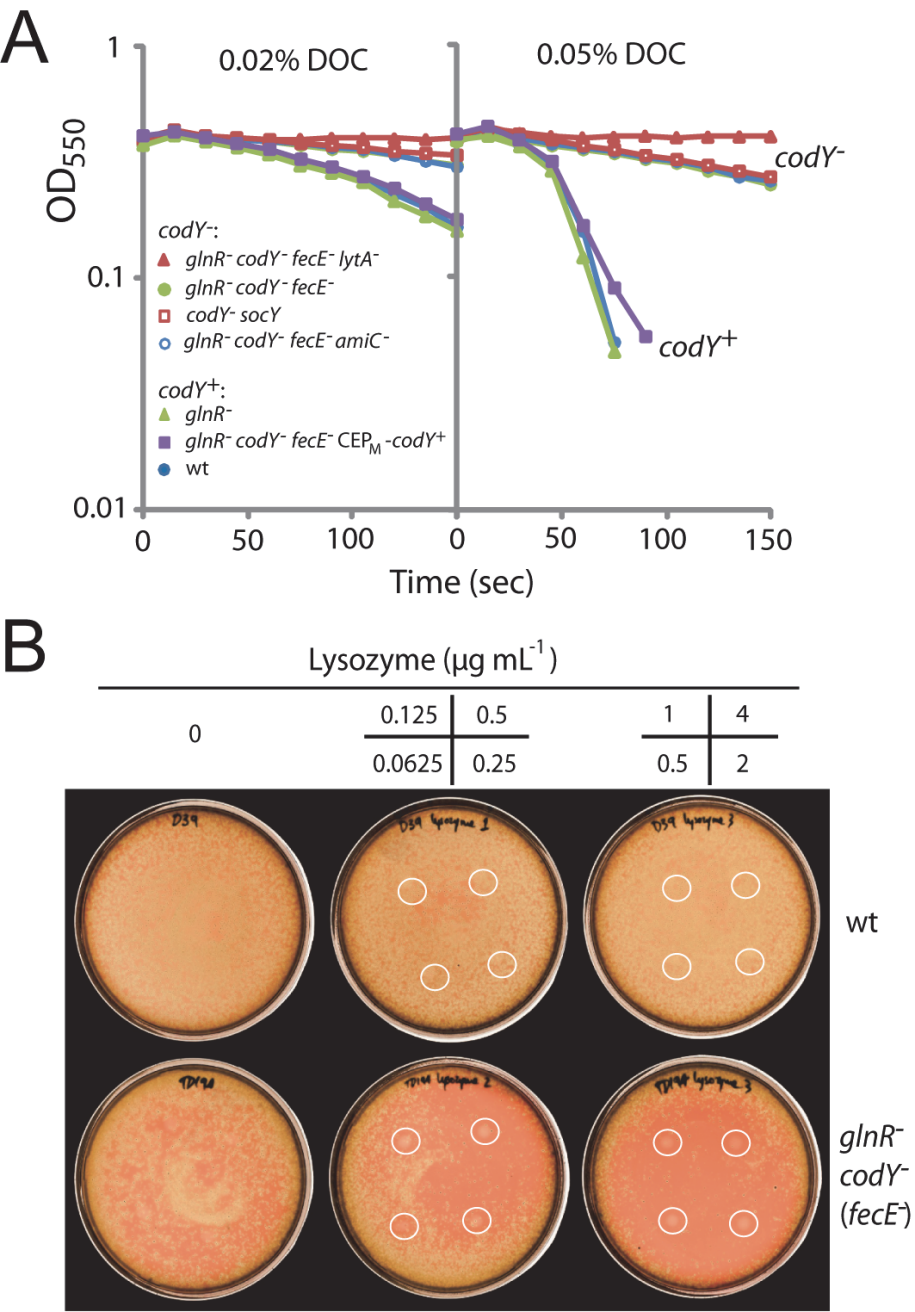
Sensitivity to DOC and lysozyme of various *codY* derivatives. (A) Sensitivity of various strains to 0.02% or 0.05% DOC. Strain identities: wt, D39; *glnR* ^-^, TD195; *codY* ^-^ *socY*, TD75; *glnR* ^-^ *codY* ^-^ (*fecE* ^-^), TK108; *glnR* ^-^ *codY* ^-^ (*fecE* ^-^) *lytA* ^-^, TD272; *glnR* ^-^ *codY* ^-^ (*fecE* ^-^) CEP_M_ *-codY* ^*+*^, TD273; *glnR* ^-^ *codY* ^-^ (*fecE* ^-^) *amiC* ^-^, TD247. (B) Sensitivity of strains to lysozyme. Lysozyme spotted onto plates in 5 μL volumes at the concentrations shown in the 4-panel grid. Spotting positions represented as white circles on the plates. Strain identities, wt; D39, *glnR* ^-^ *codY* ^-^ (*fecE* ^-^); TK108.

In fact, a puzzling observation was made when comparing the sensitivity to deoxycholate (DOC), a well-known inducer of LytA-dependent autolysis, of the *lytA* ^-^ derivative and its parent (Fig 4A). The *glnR* ^-^ *codY* ^-^ (*fecE* ^-^) parent itself appeared more resistant to DOC than the wild type. On the other hand, a *glnR* mutant behaved as wild type, while a *codY* ^-^ *socY* strain and a *glnR codY fecE amiC* mutant were also resistant to DOC (Fig 4A). Altogether, these results suggested that the inactivation of *codY* was responsible for this phenotype, a conclusion fully supported by the restoration of normal DOC sensitivity through ectopic expression of *codY* in otherwise *codY* ^-^ cells (Fig 4A).

The resistance of *codY* mutant cells to DOC appeared surprising and quite antagonistic with the rapid lysis of *glnR codY* (*fecE*) mutant cells upon plating. This apparent contradiction prompted us to examine other phenotypes, such as sensitivity to β-lactam antibiotics, which is frequently modified in pneumococcal isolates harboring peptidoglycan (PG) modifications [27–29]. A comparison of the effect of cefotaxime on various *codY* mutants and the wild type revealed that the *glnR codY* (*fecE*) mutant was much more sensitive to this antibiotic than the wild type, while *glnR* and *fecE* single mutants, a *fecE codY* double mutant and a *codY* ^-^ *socY* strain all behaved similarly to wild type (Table 1). Similarly, the *glnR codY* (*fecE*) mutant was hypersensitive to ampicillin and ceftriaxone, suggesting that sensitivity may extend to all β–lactam antibiotics (S2 Table).

**Table 1:** Sensitivity to cefotaxime of *S*. *pneumoniae* strain D39 and its *glnR*, *codY* and/or *fat*-*fec* mutant derivatives used in this study, measured as size of inhibition zone (mm).

Genotype^a^	Cefotaxime (μg mL^-1^)					
	4	1	0.25	0.062	0.016	0.0039
wt	22.0 ± 1.4	10.8 ± 0.4	1.0 ± 1.4	0	0	0
*fecE* ^-^	21.3 ± 0.4	10.5 ± 0.7	0	0	0	0
*glnR* ^-^	22.5 ± 1.4	15.0 ± 2.8	2.5 ± 3.5	0	0	0
*glnR* ^-^ *fecE* ^-^	20.8 ± 1.1	11.0 ± 0.7	0	0	0	0
*codY* ^-^ *fecE* ^-^	23.5 ± 1.4	15.8 ± 2.8	4.0 ± 0.7	0	0	0
*codY* ^-^ *fatC* ^-^ *amiC* ^-^	23.0 ± 1.4	13.5 ± 0.7	3.0 ± 1.4	0	0	0
*glnR* ^-^ *codY* ^-^ (*fecE* ^-^)	31.7 ± 0.4	27.3 ± 0.4	18.0 ± 1.4	15.3 ± 1.8	8.0 ± 0.7	0
*glnR* ^-^ *codY* ^-^ *fecE* ^-^ *amiC* ^-^	25.5 ± 0.7	17.8 ± 1.8	10.0 ± 2.8	3.0 ± 1.4	0	0

^a^ Strains used from top to bottom: TD249, TD230, TD195, TD227, TD268, TD75, TK108 and TD247.

**Fig 5:**
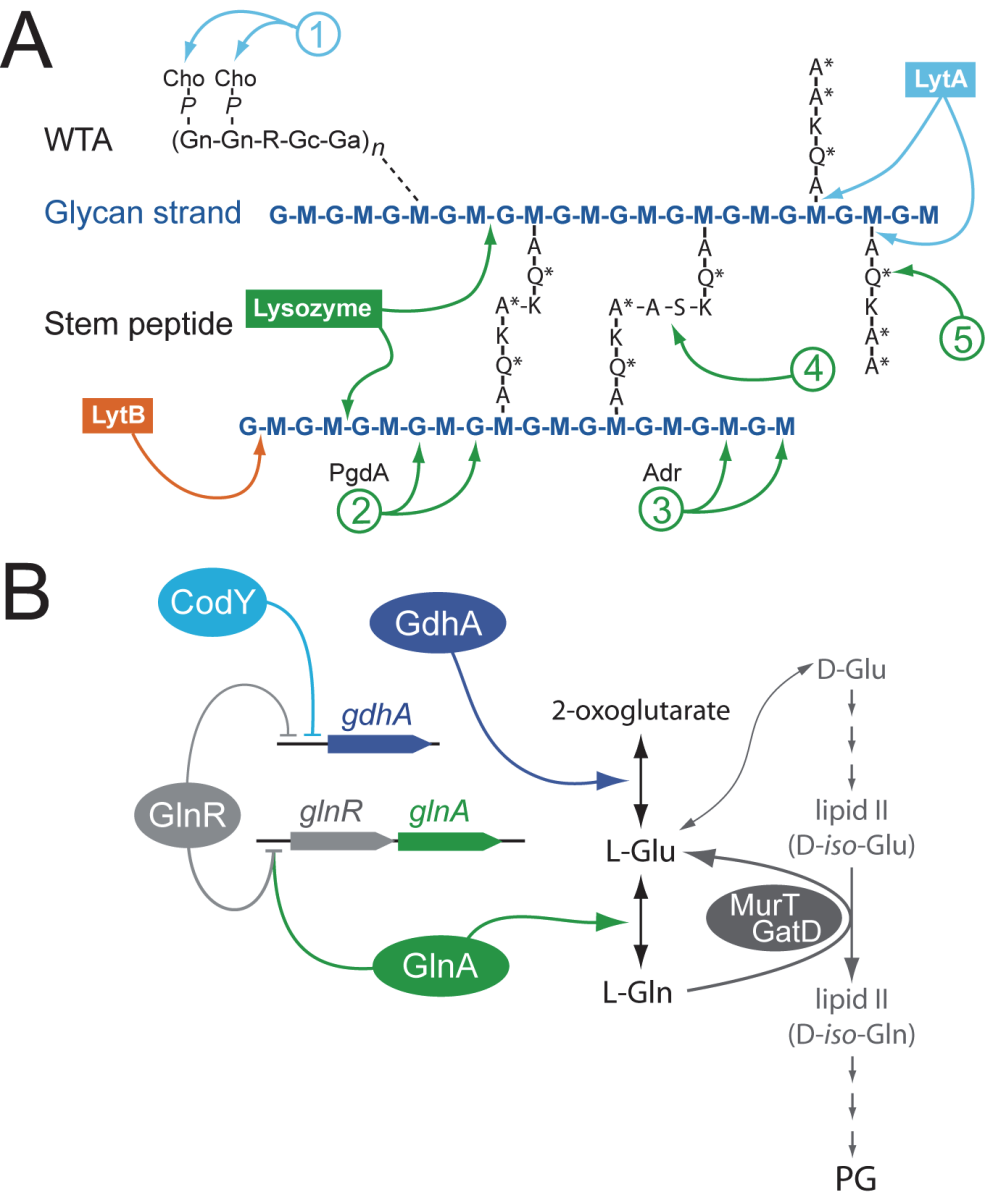
Pneumococcal cell wall composition, documented alterations and potential connections with GlnR-CodY through Glu/Gln metabolism. (A) Diagram of pneumococcal cell-wall composition, with LytA, LytB and lysozyme cleavage sites, as well as cell-wall alterations known to affect hydrolytic activities. The glycan chains in pneumococcal PG are made of alternating MurNAc (M) and GlcNAc (G) residues. WTA is composed of repeating units containing two *N*-acetylgalactosamine residues (Gn), ribitol phosphate (R), glucose (Gc) and 2-acetamido-4-amino-2,4,6-trideoxygalactose (Ga). WTA chains are linked to PG via an unknown linkage (dotted line). Each R moiety can further be alanylated, although an initially proposed a-GalpNAc substitution [48] could not be confirmed in a later work [32]. The amino acids in the stem peptide are designated in single-letter code, with an asterisk indicating d-configuration. Modifications in the cell wall are indicated by numbers: (1) decoration of WTA by phosphocholine (P-Cho); (2) deacetylation of G by PgdA; (3) *O*-acetylation of M by Adr; (4) Branching between muropeptides by MurMN; (5) Amidation at 2^nd^ stem peptide residue by MurT/GatD. (B) GlnR and CodY in the regulation of Gln/Glu metabolism of *S*. *pneumoniae*. Both regulators directly repress *gdhA*, involved in Glu synthesis. GlnR also represses *glnA*, involved in Gln synthesis, with DNA binding of GlnR to DNA shown to depend on GlnA [13]. Gln acts as a donor for amidation of the 2^nd^ stem peptide residue (see panel A) catalyzed by the MurT/GatD amidotransferase.

The β-lactam sensitivity of the *glnR* ^-^ *codY* ^-^ (*fecE* ^-^) mutant together with its colony-forming defect suggested some alteration of the cell wall. This led us to investigate its sensitivity to lysozyme, a muramidase enzyme also targeting PG (Fig 5A). While *S*. *pneumoniae* is normally resistant to lysozyme [15], the triple mutant turned out to be hypersensitive (Fig 4B). Altogether, these observations suggested that the simultaneous absence of CodY and GlnR affected cell wall physiology.

### Simultaneous inactivation of *glnR* and *codY* impacts cell wall physiology

Inactivation of both *glnR* and *codY* is expected to strongly affect Gln metabolism, as a result of full derepression of Gln synthetase and Glu dehydrogenase (Fig 5B). Since D-Gln is a key component of the PG stem peptide (Fig 5A), we hypothesized that imbalance in Gln/Glu could interfere with PG synthesis, resulting in the sensitivity phenotypes of the *glnR codY* (*fecE*) mutant. To establish whether the *glnR codY* (*fecE*) mutant displayed cell wall modifications, the composition of its PG was determined and compared with that of the wild type and other mutants used in this study. No difference was observed in the PG composition, including the percentage of unamidated peptides, the degree of stem peptide cross-linkage, and the deacetylation of muropeptides of the *glnR codY* (*fecE*) mutant compared to other strains (S2 Table and S5 Fig). In addition, we analyzed lipoteichoic acid (LTA; Materials and Methods) as a representative for pneumococcal teichoic acids, since both LTA and wall teichoic acid (WTA) have the same structure within their repeating units [30–32] and share the same biosynthesis pathway [33]. This analysis revealed no significant difference between the wild type and the *glnR codY* (*fecE*) mutant (S6 Fig).

**Fig 6:**
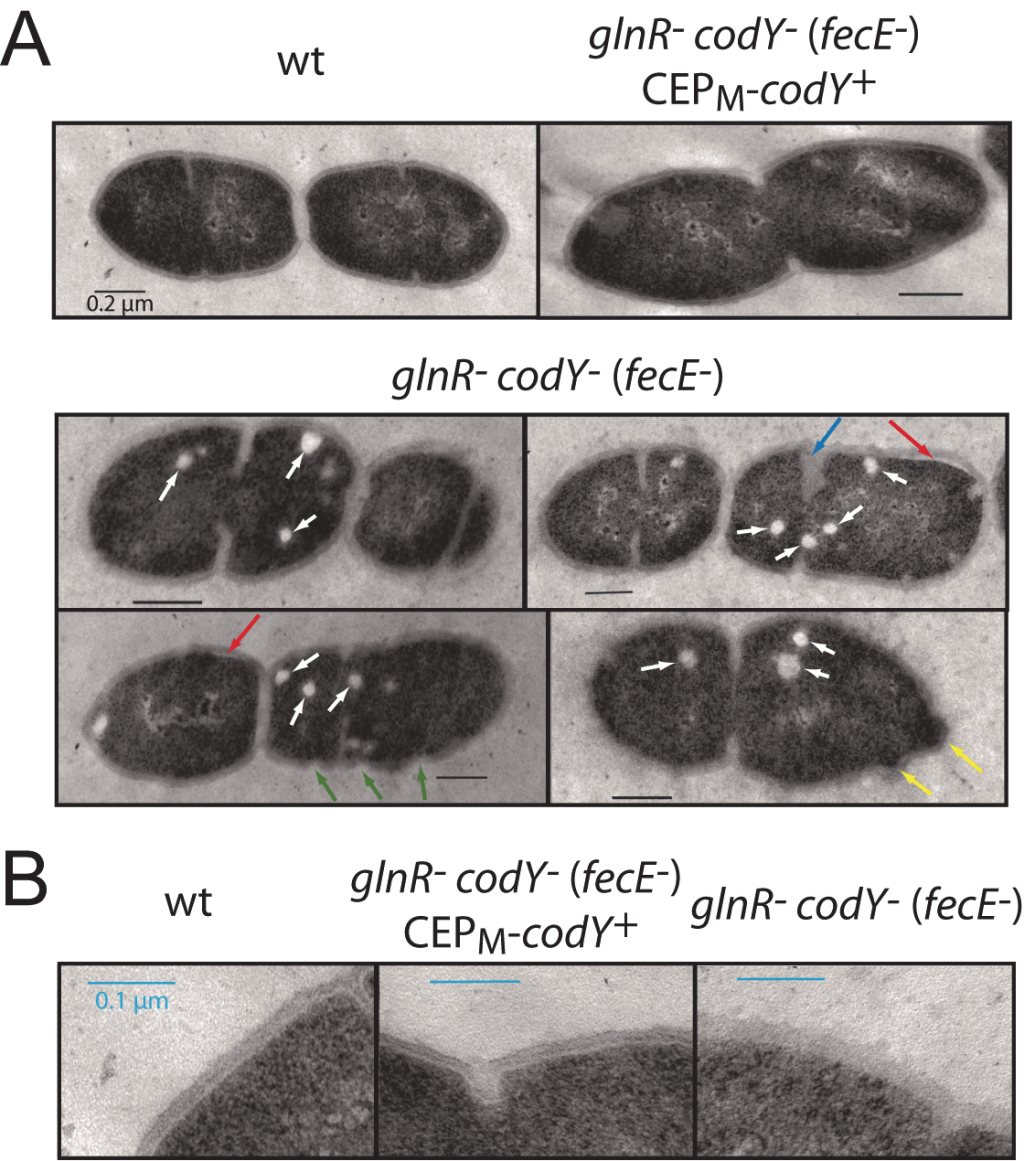
Altered cell morphology of the *glnR codY* (*fecE*) mutant visualized by TEM. (A) Visualization of wt, *glnR* ^-^ *codY* ^-^ (*fecE* ^-^) CEP_M_ *-codY* ^+^ and *glnR* ^-^ *codY* ^-^ (*fecE* ^-^) cells by TEM. Strains used, wt, D39; *glnR* ^-^ *codY* ^-^ (*fecE* ^-^) CEP_M_ *-codY* ^+^, TD273; *glnR* ^-^ *codY* ^-^ (*fecE* ^-^), TK108. Red arrows, detached cell wall; green arrows, gaps in cell wall; white arrows, circular clear bodies present within cells; blue arrow, aberrant septum formation. Black scale bars represent 0.2 μm. (B) Visualization of zoomed images of the strains in panel A focusing on cell wall. Blue scale bars represent 0.1 μm.

Despite their normal PG and LTA composition, it was possible that mutant cells had alterations in their cell wall architecture. To assess this, *glnR* ^-^ *codY* ^-^ (*fecE* ^-^) cells were visualized by transmission electron microscopy (TEM). Interestingly, these cells displayed a number of altered morphological features compared to wildtype and CEP_M_-*codY* ^*+*^ complemented *glnR* ^-^ *codY* ^-^ (*fecE* ^-^) cells (Fig 6A). Firstly, cell shape was altered, consistent with observations in S2 Fig. Secondly, the cell wall of mutant cells appeared altered and less homogenous than that of wildtype cells. Indentations into the PG were frequently observed (green arrows), as well as areas where the PG was detached from the membrane (red arrows). Thirdly, we observed oddly shaped mutant cells with small blebs on the cell surface (yellow arrows). Fourthly, septa were frequently wrongly positioned and sometimes thicker than normal (blue arrow) suggesting that the mutations affect the cell division process. In addition, the cell wall of the *glnR* ^-^ *codY* ^-^ (*fecE* ^-^) cells was less electron-dense and defined than the cell wall of wildtype or CEP_M_-*codY* ^*+*^ complemented cells (Fig 6B). Finally, we observed electrolucent foci within the mutant cells (white arrows) which were much less frequent in wildtype cells (Fig 6A). Their origin remains elusive but they do not appear to be surrounded by a membrane and may reflect the accumulation of some unknown metabolite, like fatty acids, that are extracted during the sample preparation and treatment for TEM analysis. We suggest that the morphological changes observed by TEM are caused by alterations in cell wall architecture and/or composition, explaining also the sensitivity to PG-targeting agents as a consequence of co-inactivation of *glnR* and *codY*.

## Discussion

### CodY is essential for control of iron import in pneumococci via the Fat/Fec permease

We previously showed that the global nutritional regulator CodY was essential in the human pathogen *S*. *pneumoniae* [8] and that a published *codY*::*trim* mutant [7] had a suppressing mutation inactivating the *fatC* gene [8], belonging to the *fat/fec* operon which encodes the major ferric iron ABC permease of *S*. *pneumoniae* [10]. In this study, we analyzed two further published *codY* mutants [13,14], showing that both possessed point mutations truncating the *fecE* gene, which also suppressed CodY essentiality. The detection of *fat/fec* suppressors in all three published pneumococcal *codY* mutants implies that co-inactivation of the *fat*/*fec* operon is crucial for survival of pneumococci lacking CodY. These data fully support the view that repression of iron transport is primarily responsible for the essentiality of CodY and that mutation of *codY* can only be tolerated if *fat/fec* is simultaneously inactivated to prevent iron toxicity, as previously suggested [8]. As a result, all pneumococcal *codY* mutants studied always have an obligate associated mutation inactivating the *fat/fec* operon. We refer to this mutation as the primary suppressor of CodY essentiality, crucial for survival of pneumococcal cells lacking CodY.

### Control of the Ami permease by CodY is also important though not essential

Suppressing mutations in the *amiC* gene were first identified through careful analysis of a previously published *codY* mutant [8]. *amiC* is part of the *ami* operon, which is also directly repressed by CodY and encodes the Ami oligopeptide permease. This previous study showed the *fatC* mutation in 100% of *codY* ^-^ cells but three different *amiC* mutations in the *codY* mutant population [8], consistent with a scenario in which the *fatC* mutation arose first, justifying application of the moniker ‘primary’ suppressor, and the various *amiC* mutations appeared subsequently, making them secondary suppressors. This suggested that inactivation of the Ami permease aids in tolerance of *codY* inactivation. The report that another *codY* mutant also had an inactivated *amiC* gene [14] (resulting from the very same GGA→TTGC mutation we discovered in our original analysis of *codY* essentiality [8]; Sven Hammerschmidt, personal communication) provided further support for this interpretation. However, we show that *ami* inactivation is not compulsory, as indicated by the observation that the *glnR codY* (*fecE*) mutant, which harbors no *ami* mutation, is fully viable in liquid medium (Fig 2A). The *ami* mutations can thus be viewed as accessory, aiding in but not essential for tolerance of *codY* inactivation. Nevertheless, the frequent occurrence of *ami* mutations in pneumococcal *codY* ^-^ strains suggests some level of toxicity when the *ami* operon is derepressed due to the absence of CodY.

### CodY indirectly activates pneumococcal competence by repressing *ami*

In *B*. *subtilis*, CodY plays a regulatory role on competence for genetic transformation, directly repressing the *comK* gene, encoding the transcriptional activator of competence, as well as *sfrA*, an operon important for *comK* activation [4,20,21]. Interestingly, mutation of *opp*, which encodes the Ami orthologue, resulted in loss of CodY-dependent repression of *comK* and *sfrA*, essentially activating competence [21]. Presumably, inactivation of oligopeptide import impacts CodY co-factor (i.e. branched chain amino acids) metabolism in *B*. *subtilis*.

In contrast, CodY is not known to directly repress any *com* gene in *S*. *pneumoniae*. Instead, we showed here that CodY activates competence by direct repression of *ami* (S4 Fig). As Ami antagonizes pneumococcal competence [22] through a mechanism which remains unknown, CodY thus contributes indirectly to the activation of pneumococcal competence. The opposite effects of CodY mutation on competence induction in *B*. *subtilis* and *S*. *pneumoniae* suggest different ways of responding to nutritional changes in these species. The reason for this difference could lie in the growth phases during which competence is induced in each species. In *B*. *subtilis*, competence is induced post-exponentially [34], in conditions of likely nutrient limitation. In contrast, competence in *S*. *pneumoniae* is transiently induced during exponential growth phase [35], when nutrients are generally abundant. It therefore makes sense for CodY to regulate competence induction differently in these two bacteria, which develop competence in conditions of widely different nutritional availability. Since it appears beneficial for *B*. *subtilis* to induce competence upon nutrient starvation, direct repression of competence by CodY, followed by derepression upon nutrient starvation, regulates the competence circuit to permit this. In the pneumococcus, direct repression by CodY would antagonize competence development during exponential growth, and thus no such direct regulation has been selected during evolution.

### CodY also impacts LytA-dependent autolysis

All *codY* mutants tested, including in combination with *ami* and *fec*/*fat* mutations, displayed increased resistance to DOC, an inducer of LytA-mediated autolysis, compared to wildtype cells (Fig 4A). The activity of the autolysin LytA is dependent on the decoration of WTA by choline (Cho; Fig 5A). LytA activity could therefore be affected as a consequence of altered Cho decoration. However, the putative N-acetylglucosaminidase LytB (Fig 5A), involved in daughter cell separation, is also dependent on Cho-decorated WTA for activity [36]. Although short chains can be observed in the *glnR codY* (*fecE*) mutant (S2A Fig), none of the *codY* mutants formed long chains (hundreds of cells), a hallmark of LytB inhibition [36], which suggests that LytB is at least partly active. However, it is interesting that both tested *codY* mutants had a propensity to form short chains (S2A Fig). Together with the increased resistance to DOC of all tested *codY* mutants, this observation suggests that cells lacking CodY may have reduced activity of both LytA and LytB resulting respectively in reduced lysis and increased chaining. It is possible that this is due to an alteration in the Cho-decoration of WTA, which could perturb the activity of these enzymes. However, this phenotype would be specific to all *codY* mutants, and thus remain separate from the phenotypes observed specifically in the *glnR codY* (*fecE*) mutant. The observed loss of cell viability upon plating (Fig 2C) is specific to the latter, and most probably linked to the altered sensitivity to PG-targeting agents observed.

### Co-inactivation of glnR and codY severely affects pneumococcal cell wall physiology

*glnR* ^-^ *codY* ^-^ (*fecE* ^-^) cells display a unique combination of phenotypic traits not observed with other *codY* mutants. These cells were viable in liquid medium (Fig 2A), but showed a large loss of viability when plated on solid medium (Fig 2B and 2C). Visualization of *glnR* ^-^ *codY* ^-^ (*fecE* ^-^) cells on CAT-agarose showed cells which lysed rapidly (Fig 2B), confirming the conclusion based on examination of cells fixed on microscopy slides (S2F Fig). On the other hand *codY* ^-^ *fecE* ^-^ cells appeared fully viable (Fig 2C). Inviability is therefore presumably due to derepression of both GlnR and CodY regulons. The major autolysin LytA was not responsible for the colony-forming defect observed in these cells. A specific hypersensitivity to other PG-targeting agents cefotaxime (Table 1), ampicillin, ceftriaxone (S2 Table) and lysozyme (Fig 4B) was also observed for *glnR* ^-^ *codY* ^-^ (*fecE* ^-^) cells grown on solid medium.

A number of cell wall alterations have been reported to alter pneumococcal sensitivity to certain agents targeting PG (S1 Text and Fig 5A). Potentially connected to our observations, amidation of the second residue of the stem peptide was required for efficient PG cross-linking via transpeptidase activity [31,37]. This amidation, which alters the D-*iso*-Glu to D-*iso*-Gln (Fig 5A), is dependent on the amidotransferase enzyme MurT/GatD using L-Gln as donor (Fig 5B) [37–39]. It has also been reported that inhibition of amidation reduced resistance to μ-lactam antibiotics and increased sensitivity to lysozyme in *Staphylococcus aureus* [38]. Apart from their decreased sensitivity to DOC which can be attributed to *codY* inactivation (see above), the *glnR codY* (*fecE*) mutant shared a phenotype of sensitivity to lysozyme and β-lactams with cells depleted for MurT/GatD. It was therefore tempting to speculate on the alteration in stem peptide synthesis as a cause for phenotype of the *glnR codY* (*fecE*) mutant (S1 Text). However, HPLC analysis of muropeptide composition revealed no difference in unamidated peptides as well as in the degree of PG cross-linkage between wildtype and *glnR* ^-^ *codY* ^-^ (*fecE* ^-^) cells (S2 Table), providing no support to this hypothesis. In addition, while our analysis could not detect *O*-acetylation, a potential mechanism for lysozyme resistance (Fig 5A, number 3), as (most of) the *O*-acetyl groups are lost during routine sample preparation, we were able to detect deacetylated muropeptides (another mechanism for lysozyme resistance; Fig 5A, number 2). However, there was no dramatic difference in levels of deacetylated muropeptides in the *glnR codY* (*fecE*) mutant that could explain its lysozyme sensitivity.

Despite this, visualization of cells by TEM uncovered stark differences in cell wall morpohology between wildtype and *glnR codY* (*fecE*) mutant cells, with less dense cell wall observed in the mutant (Fig 6B), along with regions where the cell wall appears disrupted or detached from the cell (Fig 6A). These results strongly suggest that the *glnR codY* (*fecE*) mutant has altered PG, which we propose results in the increased sensitivity to PG-targeting agents. However, both the exact physical nature of the alterations observed and the underlying mechanism(s) which can be indirect, owing to alterations in central metabolism in the absence of both CodY and GlnR regulators, remain to be established.

### Connections between iron toxicity, cell wall physiology and Ami

While *glnR* ^-^ *codY* ^-^ (*fecE* ^-^) cells display a unique phenotype among *codY* mutants, the observation that inactivation of *ami* restored viability of these cells (Fig 3B) indicates that the very same combination of suppressing mutations allowing survival of *codY* mutant cells (i.e. *ami* and *fat*/*fec*) can compensate for the absence of both GlnR and CodY. The nature of the compound(s) imported via the Ami permease which is ‘toxic’ for the cell remains unknown. In view of the observation that an Ami homologue allowed use of heme as an iron source by *Escherichia coli* [40], we previously suggested that Ami could similarly contribute to iron toxicity in *S*. *pneumoniae* [8]. However, no direct support for this hypothesis could be obtained since we observed that plating with or without horse blood (i.e. in the presence of widely different heme concentrations) did not alter survival of *glnR* ^-^ *codY* ^-^ (*fecE* ^-^) cells (data not shown). In addition, even if iron toxicity proved to be responsible for inviability of *codY* and *codY glnR* mutants, the link between cell wall physiology and iron toxicity would remain unclear. Alternatively, Ami toxicity may not be connected with iron toxicity but somehow linked to the recycling of peptidoglycan during growth. In *Escherichia coli*, peptidoglycan recycling is known to rely primarily on the AmpG transporter, which is specific for uptake of anhydromuropeptides, whilst the oligopeptide permease Opp (the orthologue of Ami) has only a minor role in uptake of cell wall derived peptides. In contrast, with Gram-positives lacking AmpG, Ami-like permeases may constitute the major cell wall recycling pathway [41]. It is thus possible that Ami is responsible for peptidoglycan recycling in *S*. *pneumoniae*, and altered recycling in the absence of *codY* is toxic on solid medium. Alternatively, the effect of *ami* inactivation could be indirect, resulting from the reduced growth rate conferred by *ami* mutations. A reduction in growth rate could for example compensate for metabolism jamming by allowing more time to the cell to build the envelope.

### Concluding remarks

The genetic dissection of previously published *codY* mutants allowed us to demonstrate here that the primary suppressing mutation necessary to tolerate absence of CodY is inactivation of the *fat/fec* iron permease operon. This strongly suggests that unregulated iron import is toxic to *codY* ^-^ pneumococci. Furthermore, we showed that a *glnR codY* (*fecE*) mutant was only partly viable on solid medium and displayed a unique phenotype among *codY* mutants, suggestive of altered cell wall physiology, a conclusion fully supported by TEM analysis of mutant cells. While increasing resistance to PG-targeting antibiotics such as β-lactams is problematic for treatment of pneumococcal infections, hypersensitivity of this mutant to PG-targeting agents suggests GlnR and CodY as novel therapeutic targets. Simultaneous targeting of these important nutritional regulators would render pneumococci hypersensitive to both iron and classic PG-targeting antibiotics, and could thus be useful in the constant fight against this debilitating pathogen.

## Materials and Methods

### Bacterial strains, growth and transformation conditions

*S*. *pneumoniae* strain growth and transformation were carried out as described [8]. Strain and primer information can be found in S1 Table. Unless stated, recipient strains were rendered *hex* ^-^ by insertion of the *hexA*::*ermAM* cassette as previously described [42], to negate any effect of the mismatch repair system on transformation efficiencies via rejection of mismatched DNA in transformation heteroduplexes [43]. Antibiotics were used at the following concentrations; trimethoprim 200 μg mL^-1^, erythromycin 2 μg mL^-1^, kanamycin 250 μg mL^-1^, streptomycin 200 μg mL^-1^, methotrexate μg mL^-1^. Previously described cassettes used for transfer were *glnR*::*kan22* ^C^ [17] and *ΔcodY*::*trim* [13]. Growth curves were followed in a 96-well Spectrophotometer (ThermoScientific) with OD_492_ readings every 10 min after 1/100 dilution of cultures grown to OD 0.2 into a final volume of 300 μL. Doubling times were calculated as the time required to observe a doubling of OD during the fastest phase of exponential growth. As a result, this figure does not take into account any lag phase prior to active growth that can be seen in certain conditions with different mutants.

Competence was monitored as previously described [44] in pH gradients in C+Y medium on strains containing the *ssbB-luc* transcriptional fusion between the promoter of the single-stranded DNA-binding protein SsbB, which is induced during competence [4], and the firefly luciferase gene *luc*, the fusion thus reporting on competence through light emission by luciferase [45]. Briefly, cells were grown in conditions where competence does not spontaneously develop (2 mL C+Y pH 6.6) to OD_550_ 0.11, pelleted and resuspended in 1 mL C+Y. 100 μL aliquots were diluted 1/10 in C+Y, and 30 μL added to 250 μL C+Y culture of desired pH and 20 μL luciferin (3 mg mL^-1^) in microtitre plate wells. Emission of light and OD_492_ values were followed throughout growth.

### Genome sequencing

Genome sequencing of the TK108 strain was carried out by LGC genomics. The resulting sequence was aligned to the D39 genome present in the NCBI database (NCTC 7466), and point mutations, insertions and deletions were identified by LGC genomics.

### Transfer of *fecE* ^-^ point mutation without selection

Transfer of the *fecE* ^-^ point mutation of TK108 into a D39 recipient strain was achieved without selection. The mutation was amplified from TK108 by PCR with 500 bp flanking DNA on either side. The PCR product (~1,000 bp) was purified using a purification kit (QIAGEN), and transformed into the TD195 strain to create strain TD227. Briefly, 100 μL pre-competent TD195 cells were resuspended in 900 μL C+Y medium with 100 ng μL^-1^ CSP. Cells were incubated at 37°C for 10 min to induce competence, and 1 μg of *fecE* ^-^ PCR product was added to 100 μL competent culture. This transformation mixture was incubated at 30°C for 20 min, before addition of 1.4 mL C+Y medium, and further incubation at 37°C for 4 h to allow correct integration of the mutation and cell separation. Cells were then plated on CAT agarose plates + 5% defibrinated horse blood (Biomérieux), and incubated overnight at 37°C. Colonies are then picked and analyzed by PCR and sequencing to identify those that had acquired the *fecE* ^-^ point mutation, before sub-cloning to ensure a pure *fecE* mutant culture.

### Mutation of CodY binding site in *ami* promoter

In order to mutate the *CYbs* in the *ami* promoter region, a fragment of DNA of 1,000 bp, with the *CYbs* (AATTTTCAGAATATT) replaced by a sequence containing an *Nco*I restriction site (GCTAGGGATCCGCTA) and flanked on either side by 500 bp of DNA was synthesized (Genscript). This fragment was transformed into *S*. *pneumoniae* strains, as described for *fecE* ^-^, in the absence of antibiotic selection, and transformant clones were identified by PCR of the *ami* promoter and restriction by *Nco*I.

### Microscopy

For static images of cells analyzed in S2 and S3 Figs, cells were grown in C+Y medium to an OD_492_ of 0.2, and 2 μL of these cultures was spotted onto a microscope slide. A polylysin-coated coverslip was placed on top to fix the cells in position. Images were captured and processed using the Nis-Elements AR software (Nikon). Analysis of cell dimensions was carried out using the MATLAB-based open source software MicrobeTracker [46]. Cell contours were obtained using the *alg4 spneumoniae3* algorithm implemented in MicrobeTracker, a derivative of *alg4 ecoli2* with parameters spliltTreshold, joindist and joinangle refined to fit the shape of *S*. *pneumoniae*.

Time-lapse microscopy was carried out as previously described [47], with modifications. Briefly, pneumococcal precultures were grown in liquid CAT medium at 37°C to an OD_492_ of 0.2, and 2 μL of these cultures was spotted onto a microscope slide containing a slab of 1.2% CAT agarose before imaging. Images were captured at 5 min intervals for 6 hours and processed using the Nis-Elements AR software (Nikon).

### Sensitivity tests

Sensitivity to cefotaxime, ampicillin, ceftriaxone and lysozyme was measured by growing strains to OD_550_ 0.2 in C+Y medium, followed by plating ~400–600 cells on CAT-agarose supplemented with 5% horse blood (Biomérieux). After drying, the inhibitory agent was spotted onto these plates in 5 μL volumes (4 spots per plate) at 4-fold dilutions starting at 4 μg mL^-1^ (see Table 1). Plates were incubated at 37°C overnight and inhibition zones were measured. Sensitivity to DOC was measured by growing strains to OD_550_ ~0.4 in C+Y medium, followed by addition of 0.02% or 0.05% DOC to 1 mL of culture. OD_550_ readings were taken every 15 sec for 150 sec.

### Isolation and analysis of peptidoglycan

Peptidoglycan was isolated as previously published [31]. Digestion of peptidoglycan with cellosyl (kindly provided by Hoechst, Germany) yielded muropeptides, which were reduced with sodium borohydride and analyzed by high-pressure liquid chromatograhy as published [31].

### Isolation and analysis of LTA

LTA from *S*. *pneumoniae* D39 and TK108 (*glnR* ^-^ *codY* ^-^ *(fecE* ^-^ *)*) was isolated as previously described [32]. Pneumococci were grown in 5 L cultures THY medium. Samples were dissolved in a water/propan-2-ol/7 M triethylamine/acetic acid mixture (50:50:0.06:0.02, v/v/v/v) and analysed using a APEX Qe Fourier-Transform Ion Cyclotron Resonance Mass Spectrometer (Bruker Daltonics, USA) equipped with the Triversa Nanomate (Advion, USA) as ion source applying a spray voltage of -1.1 kV. Mass spectra were recorded in negative-ion mode in the broadband acquisition mode, the mass scale was externally calibrated with glycolipids of known structure, and spectra were smoothed, baseline corrected, and charge deconvoluted.

### TEM

Wild type and *glnR* ^-^ *codY* ^-^ (*fecE* ^-^) cells were exponentially grown at 37°C in C+Y medium. Samples were then collected, centrifuged and fixed overnight with 5% glutaraldehyde in 0.1 M cacodylate buffer (pH 7.5) at 4°C. Postfixation with 1% osmium tetroxide in cacodylate buffer was carried out for 1 h at room temperature. These fixed cells were dehydrated using a graded series of ethanol and embedded in LR White at 60°C for 48 h. Ultrathin sections (50 nm) were obtained using a Leica UC7 microtome and were counterstained with uranyl acetate and lead citrate (Reichert Ultrostainer, Leica, Germany). Samples were examined with a Philips CM120 transmission electron microscope equipped with a Gatan Orius SC200 CCD camera.

## Supporting Information

S1 Fig Growth curves of *codY* mutants in different media. (A-C) Growth curves of strains in CAT, TSB and THY media respectively. After initial growth to identical cellular densities (OD 0.2) in medium, cells were diluted 1/100 in 300 μL final volume of appropriate medium in microtitre plate and OD_492_ readings taken every 10 min for 600 min. Strain identities, wt, D39; *glnR* ^-^ *codY* ^-^ (*fecE* ^-^), TK108; *codY* ^-^ *socY*, TD75.
(TIF)

S2 Fig Exploring cell morphology by microscopy. (A) Wildtype, *glnR* ^-^ *codY* ^-^ (*fecE* ^-^) and *codY* ^-^ *socY* cells observed on polylysin slides. Strains used, wt, D39; *glnR* ^-^ *codY* ^-^ (*fecE* ^-^), TK108; *codY* ^-^ *socY*, TD75. (B) Length of cells observed on polylysin slides. Data represented as percentage of cell population fitting into different length fractions. It is of note that every cell in a chain (panel G) was nevertheless treated as a single cell; therefore chaining should have had no influence on the calculation of the cell length parameter. The vertical dashed red line represents the average value (indicated above the line) of wildtype cells. Strains used as in panel A. (C) Width of cells observed on polylysin slides. Strains and analysis as in panel B. (D) Area of cells observed on polylysin slides. Strains and analysis as in panel B. (E) Hypothetical volume of cells observed on polylysin slides. Hypothetical value calculated from other values of cell dimension. Strains and analysis as in panel B. (F) Mean number of individual cells counted per image. Strains as in panel A. (G) Percentage of cells per image which form part of chains. Strains as in panel A.
(TIF)

S3 Fig Exploring cell morphology by microscopy in control strains. (A) Length of cells observed on polylysin slides. Data represented as percentage of cell population fitting into different length fractions. Strains used, wt, D39; *glnR* ^-^ *fecE* ^-^, TD227; *glnR* ^-^ *codY* ^-^ (*fecE* ^-^) CEP_M_-*codY* ^*+*^, TD273. (B) Width of cells observed on polylysin slides. Strains, cells and analysis as in panel A. (C) Area of cells observed on polylysin slides. Strains, cells and analysis as in panel A. (D) Hypothetical volume of cells observed on polylysin slides. Strains, cells and analysis as in panel A. Hypothetical value calculated from other values of cell dimension. (E) Mean number of cells counted per image. Strains as in panel A. (F) Percentage of cells per image which form part of chains. Strains as in panel A.
(TIF)

S4 Fig Monitoring spontaneous competence development in connection with *codY* inactivation. (A) Development of competence in wt background monitored by following expression of *ssbB-luc* transcriptional fusion. Competence development (RLU/OD) was plotted against growth (OD_492_) in a gradient of varying starting pHs. Strain used, TD259. (B) Development of competence in a *fecE* mutant. Strain used TD263. Experimental information and figure layout as in panel A. (C) Development of competence in a *fecE codY* double mutant. Strain used TD265. Experimental information and figure layout as in panel A. (D) Derepression of *ami* antagonizes competence revealed by monitoring competence development in an *ami* ^*CYbs0*^ mutant. Strain used TD260. Experimental information and figure layout as in panel A. (E) Development of competence in a *fecE ami* ^*CYbs0*^ double mutant. Strain used TD264. Experimental information and figure layout as in panel A.
(TIF)

S5 Fig HPLC analysis of muropeptide composition. Peptidoglycan was digested with the muramidase cellosyl and the resulting muropeptides were reduced with sodium borohydride and analysed by high-pressure liquid chromatography. Strains used (from top to bottom: R6, TD249, TD227, TK108, TD247 and TD75) are indicated on the right side.
(TIF)

S6 Fig Analysis of pneumococcal LTA. (A) Section (5500–10500 Da) of the charge deconvoluted ESI-FT-ICR-MS spectrum of pnLTA isolated from strain D39 (wt, black) and TK108 (*glnR* ^-^ *codY* ^-^ (*fecE* ^-^), red). (B) Enlarged image of the ion cluster with highest intensity (8450–8750 Da). Differences in the signal pattern of the two strains are caused by varying percentage of sodium adduct ion cluster, as indicated. For a clear visualization of mass differences, the most intensive peak of an ion cluster has been chosen instead of the monoisotopic peak.
(TIF)

S1 Table Strains, plasmids and primers used in this study. (DOCX)

S2 Table Sensitivity to ampicillin and ceftriaxone of *S*. *pneumoniae* strain D39 and its *glnR*, *codY* (*fecE*) derivative TK108. (DOCX)

S3 Table Peptidoglycan composition of wildtype and mutant strains. (DOCX)

S1 Text Supporting Discussion. (DOCX)
