# Supplementary figures and images for: Co-Inactivation of GlnR and CodY Regulators Impacts Pneumococcal Cell Wall Physiology
Source: PLoS One. 2015 Apr 22;10(4):e0123702. doi: 10.1371/journal.pone.0123702 (PMC4406557; doi:10.1371/journal.pone.0123702)

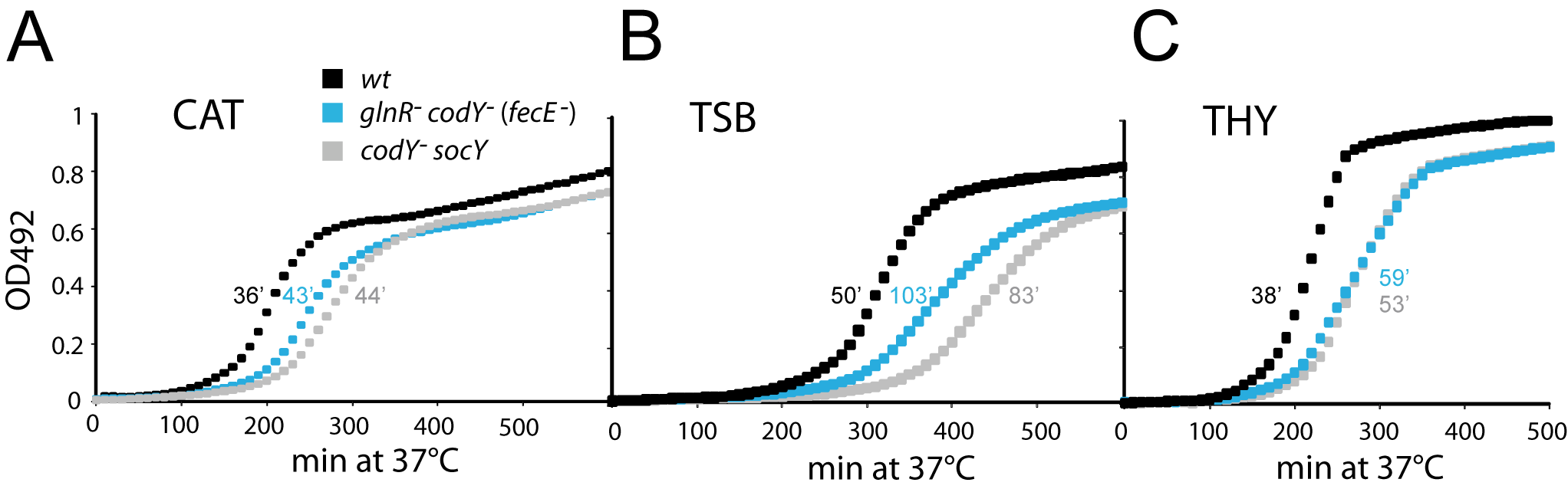

Supplement: S1 Fig — (A-C) Growth curves of strains in CAT, TSB and THY media respectively. After initial growth to identical cellular densities (OD 0.2) in medium, cells were diluted 1/100 in 300 μL final volume of appropriate medium in microtitre plate and OD492 readings taken every 10 min for 600 min. Strain identities, wt, D39; glnR - codY - (fecE -), TK108; codY - socY, TD75. (TIF) [file pone.0123702.s001.tif]

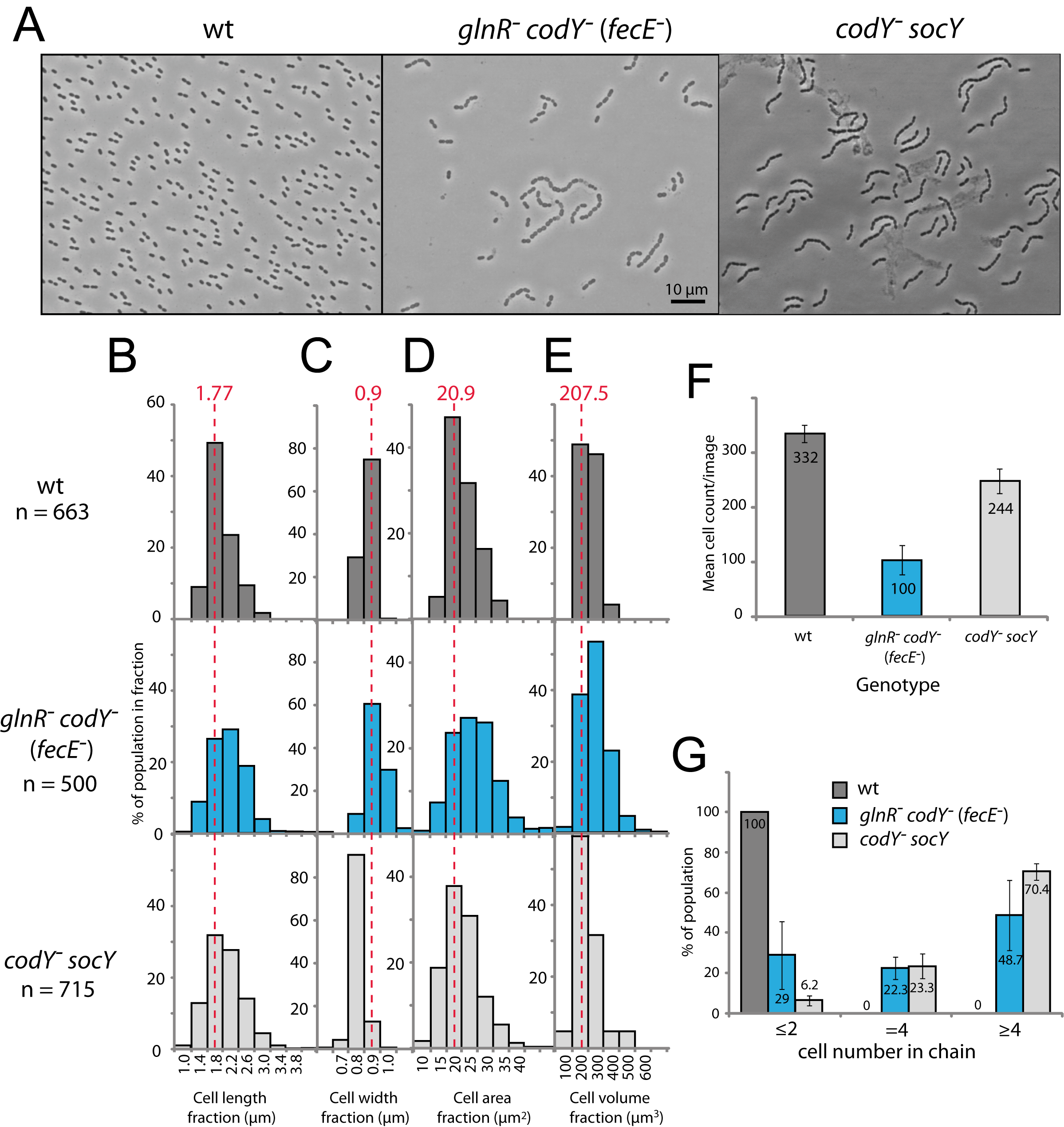

Supplement: S2 Fig — (A) Wildtype, glnR - codY - (fecE -) and codY - socY cells observed on polylysin slides. Strains used, wt, D39; glnR - codY - (fecE -), TK108; codY - socY, TD75. (B) Length of cells observed on polylysin slides. Data represented as percentage of cell population fitting into different length fractions. It is of note that every cell in a chain (panel G) was nevertheless treated as a single cell; therefore chaining should have had no influence on the calculation of the cell length parameter. The vertical dashed red line represents the average value (indicated above the line) of wildtype cells. Strains used as in panel A. (C) Width of cells observed on polylysin slides. Strains and analysis as in panel B. (D) Area of cells observed on polylysin slides. Strains and analysis as in panel B. (E) Hypothetical volume of cells observed on polylysin slides. Hypothetical value calculated from other values of cell dimension. Strains and analysis as in panel B. (F) Mean number of individual cells counted per image. Strains as in panel A. (G) Percentage of cells per image which form part of chains. Strains as in panel A. (TIF) [file pone.0123702.s002.tif]

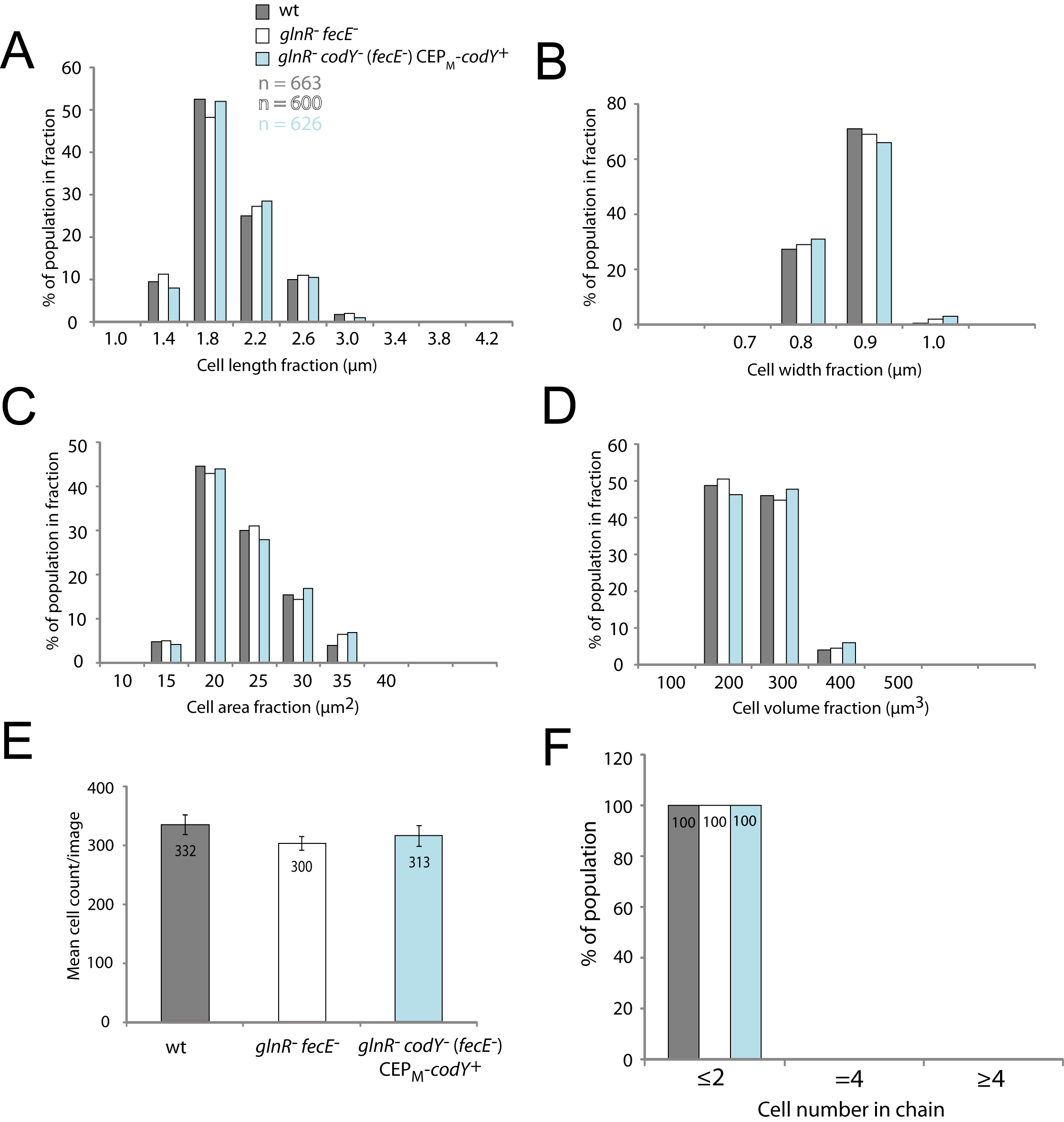

Supplement: S3 Fig — (A) Length of cells observed on polylysin slides. Data represented as percentage of cell population fitting into different length fractions. Strains used, wt, D39; glnR - fecE -, TD227; glnR - codY - (fecE -) CEPM-codY +, TD273. (B) Width of cells observed on polylysin slides. Strains, cells and analysis as in panel A. (C) Area of cells observed on polylysin slides. Strains, cells and analysis as in panel A. (D) Hypothetical volume of cells observed on polylysin slides. Strains, cells and analysis as in panel A. Hypothetical value calculated from other values of cell dimension. (E) Mean number of cells counted per image. Strains as in panel A. (F) Percentage of cells per image which form part of chains. Strains as in panel A. (TIF) [file pone.0123702.s003.tif]

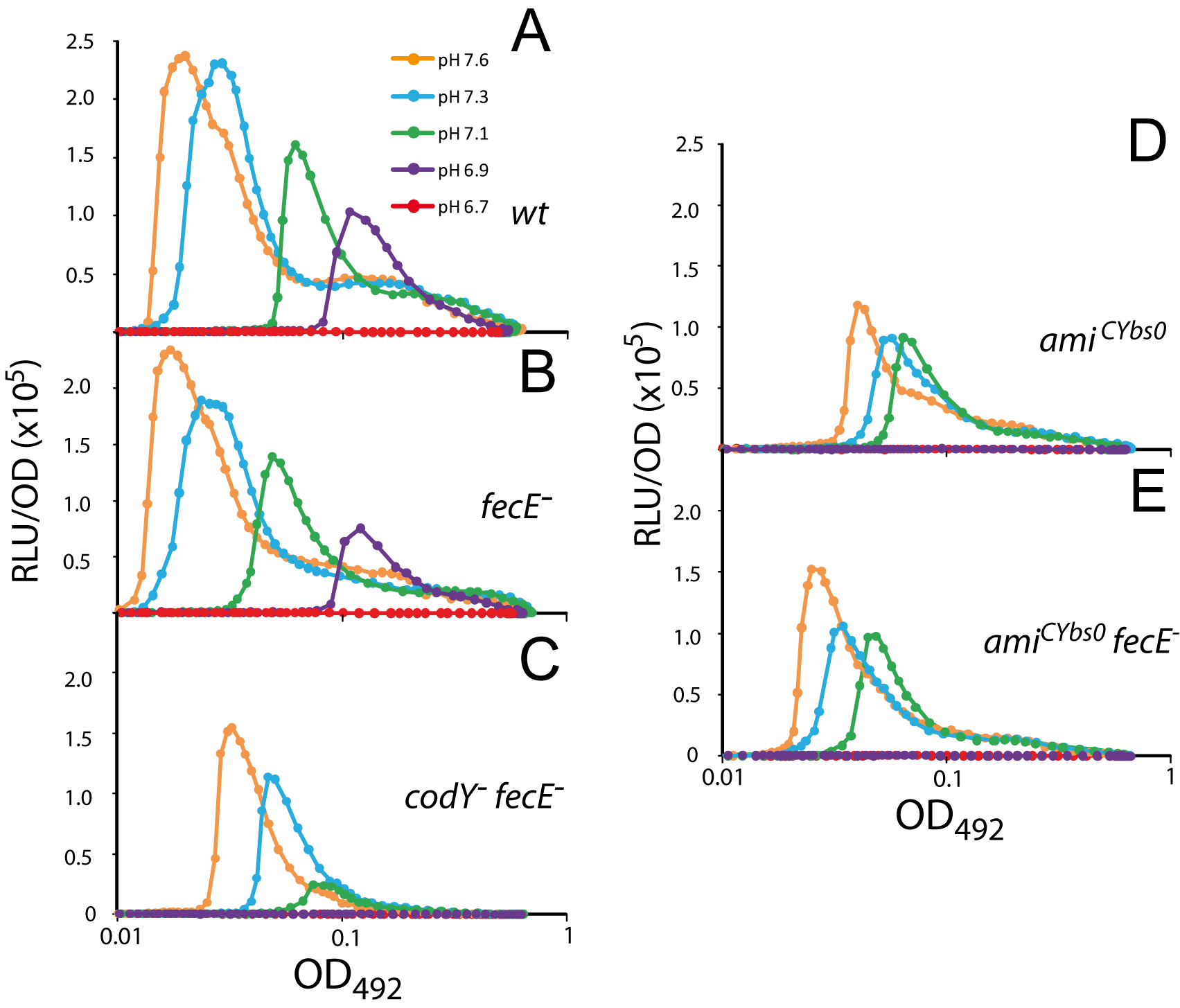

Supplement: S4 Fig — (A) Development of competence in wt background monitored by following expression of ssbB-luc transcriptional fusion. Competence development (RLU/OD) was plotted against growth (OD492) in a gradient of varying starting pHs. Strain used, TD259. (B) Development of competence in a fecE mutant. Strain used TD263. Experimental information and figure layout as in panel A. (C) Development of competence in a fecE codY double mutant. Strain used TD265. Experimental information and figure layout as in panel A. (D) Derepression of ami antagonizes competence revealed by monitoring competence development in an ami CYbs0 mutant. Strain used TD260. Experimental information and figure layout as in panel A. (E) Development of competence in a fecE ami CYbs0 double mutant. Strain used TD264. Experimental information and figure layout as in panel A. (TIF) [file pone.0123702.s004.tif]

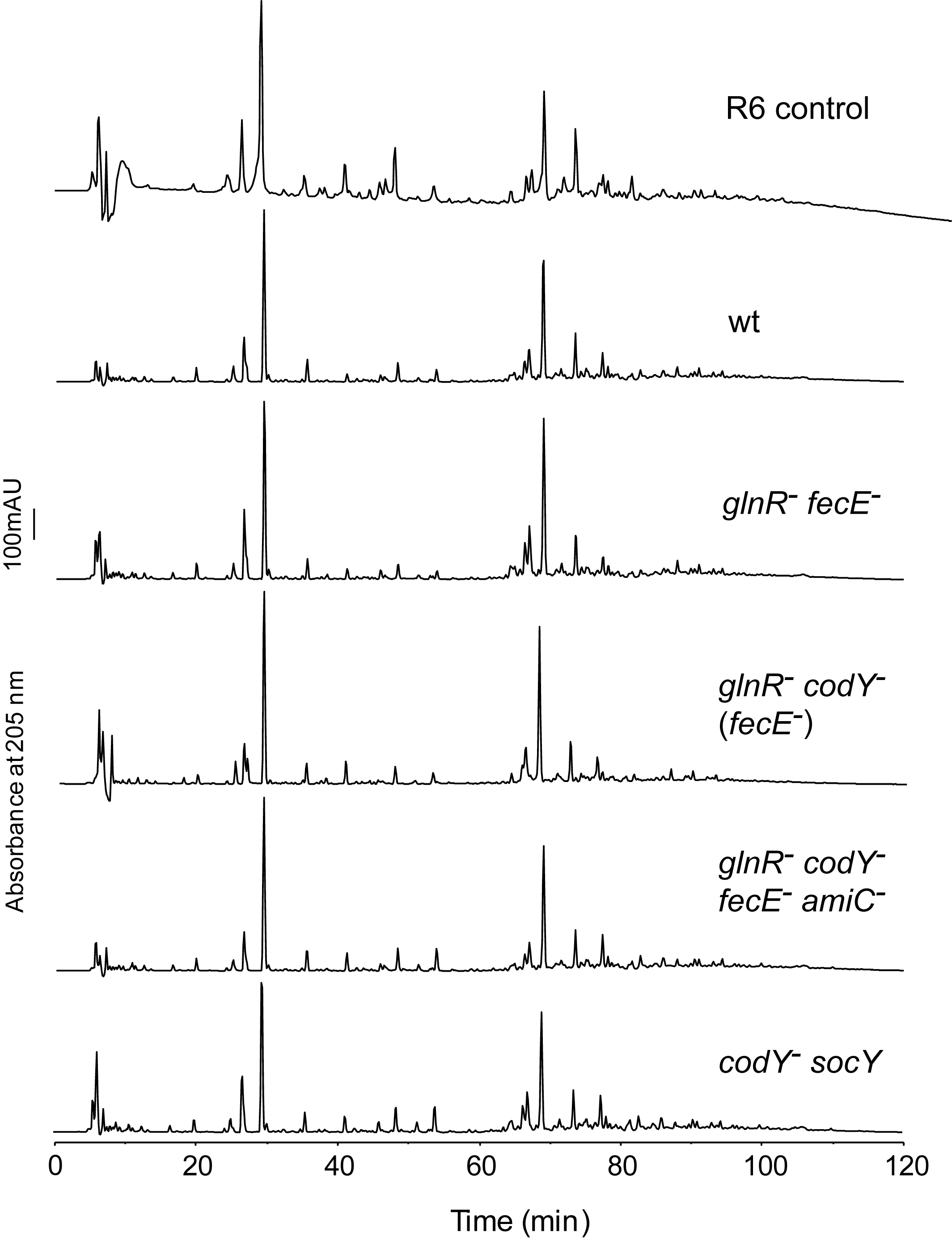

Supplement: S5 Fig — Peptidoglycan was digested with the muramidase cellosyl and the resulting muropeptides were reduced with sodium borohydride and analysed by high-pressure liquid chromatography. Strains used (from top to bottom: R6, TD249, TD227, TK108, TD247 and TD75) are indicated on the right side. (TIF) [file pone.0123702.s005.tif]

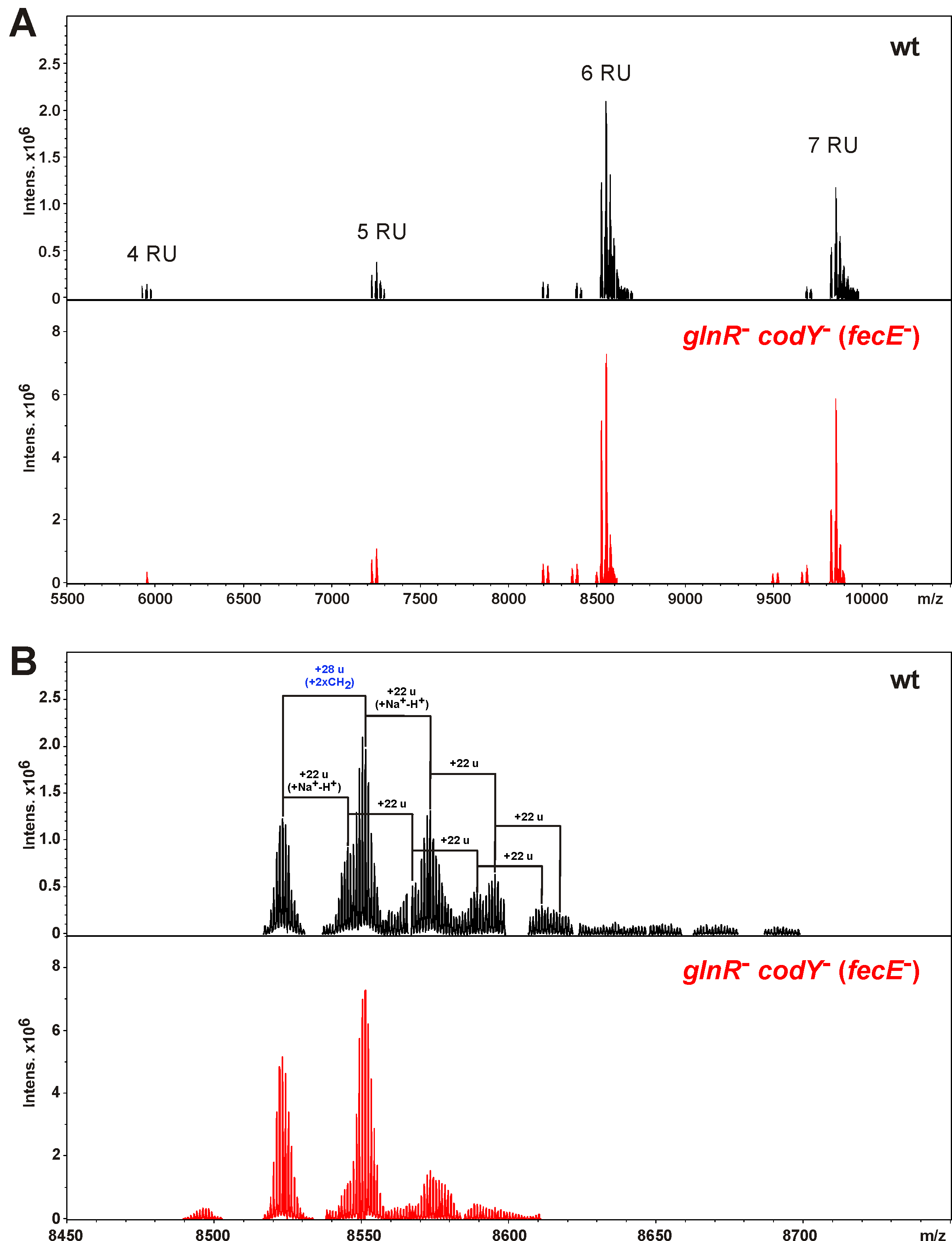

Supplement: S6 Fig — (A) Section (5500–10500 Da) of the charge deconvoluted ESI-FT-ICR-MS spectrum of pnLTA isolated from strain D39 (wt, black) and TK108 (glnR - codY - (fecE -), red). (B) Enlarged image of the ion cluster with highest intensity (8450–8750 Da). Differences in the signal pattern of the two strains are caused by varying percentage of sodium adduct ion cluster, as indicated. For a clear visualization of mass differences, the most intensive peak of an ion cluster has been chosen instead of the monoisotopic peak. (TIF) [file pone.0123702.s006.tif]
