# Supplementary material for: Co-Inactivation of GlnR and CodY Regulators Impacts Pneumococcal Cell Wall Physiology
Source: PLoS One. 2015 Apr 22;10(4):e0123702. doi: 10.1371/journal.pone.0123702 (PMC4406557; doi:10.1371/journal.pone.0123702)
Supplement: S1 Table — (DOCX) [file pone.0123702.s007.docx]

| Table S1 Strains, plasmids and primers used in this study. | |  |
| --- | --- | --- |
|  |  |  |
| ***S. pneumoniae*  strain** | **Genotype**^a^**/Description** | **Source/Reference** |
|  |  |  |
| D39 | Serotype 2 strain, cps2 | NCTC 7466 |
| R246 | R800 *hexA*::*ermAM*; Ery^R^ | [1] |
| R800 | R6 derivative strain | [2] |
| R2437 | R800 ∆*comC*, CEP_M_-*codY* (resulting duplication is *codY^+/+^*), *rpsL41*;  Kan^R^, Sm^R^ | [3] |
| R3154 | R800 ∆*comC*, *glnR*::*kan^22^*^C^, *rpsL41*; Kan^R^, Sm^R^ | [4] |
| TD75^b^ | D39 *codY*::*trim*, *socY*^c^; Trim^R^, Mtx^R^ | [5] |
| TD80 | D39 ∆*cps*::*kan*, *codY*::*trim*, *socY*^c^, *rpsL41*; Kan^R^, Sm^R^, Trim^R^, Mtx^R^ | [3] |
| TD81 | TD80 but *codY*::*trim* replaced by *codY*::*spc*^3A^; Kan^R^, Sm^R^, Spc^R^, Mtx^R^ | [3] |
| TD141 | D39 ∆*cps*::*kan*, *hexA*::*ermAM*, *fat*::*cat*^23C^ ; Kan^R^, Ery^R^, Cm^R^ | This study |
| TD142 | TD141 but *amiC9*; Kan^R^, Ery^R^, Cm^R^, Mtx^R^ | This study |
| TD195 | TK102 but *hexA*::*ermAM*; Ery^R^ | This study |
| TD196 | TK108 but *rpsL41*; Trim^R^, Sm^R^ | This study |
| TD198 | D39 *hexA*::*ermAM*; Ery^R^ | This study |
| TD212 | TD195 but *fatC*::*cat*^23C^; Ery^R^, Cm^R^ | This study |
| TD227 | TD195 but *fecE^-^*; Ery^R^ | This study |
| TD228 | TD227 but *amiC9*; Ery^R^, Mtx^R^ | This study |
| TD230 | TD198 but *fecE^-^*; Ery^R^ | This study |
| TD247 | TK108 but *amiC9*; Trim^R^, Mtx^R^ | This study |
| TD249 | D39 but *hexA*::*ermAM*; Ery^R^ | This study |
| TD250 | TD75 but *hexA*::*spc*; Trim^R^, Mtx^R^, Spc^R^ | This study |
| TD252 | TK108 but *hexA*::*spc*; Trim^R^, Spc^R^ | This study |
| TD259 | D39 *hexA*::*spc*, *ssbB-luc*; Spc^R^, Cm^R^ | This study |
| TD260 | TD259 but *ami^CYbs0^*^d^; Spc^R^; Cm^R^ | This study |
| TD263 | TD230 but *ssbB-luc*; Ery^R^, Cm^R^ | This study |
| TD264 | TD263 but ami*^CYbs0^*^d^; Ery^R^, Cm^R^ | This study |
| TD265 | TD262 but *ssbB-luc*; Ery^R^, Trim^R^, Cm^R^ | This study |
| TD268 | D39 *codY*::*trim* *fecE*^-^; Trim^R^ | This study |
| TD272 | TK108 but *lytA*::*cat* ; Trim^R^ Cm^R^ | This study |
| TD273 | TK108 but CEP_M_-*codY;* Trim^R^, Kan^R^ | This study |
| TK102 | D39 *glnR*stop | [6] |
| TK108 | TK102 but *codY*::*trim* (and *fecE^-^*, as shown in this study); Trim^R^ | [6]; this study |
| R3701 | R2437 but ectopic *codY* inactivated by *mariner* insertion *spc*^3A^ (*codY^+/spc^*);  Kan^R^, Sm^R^, Spc^R^ | This study |
| **Plasmid** | **Description** | **Source/reference** |
| pCC1-*ami^CYbs0^*^d^ | pCC1 plasmid containing CodY binding site (*CYbs*) of *ami* promoter mutated to possess *Bam*HI, and 500 bp of flanking DNA on either side to allow integration in the absence of selection | This study |
| **Primer** | **Sequence, gene, position**^e^ | **Source/reference** |
| CJ156 | GATCATTGAGAGTGTGGGTGGCTTC, *fecE*, -35 | This study |
| CJ157 | ACTGAAGGTTCTCTAACTTCTTGAGCTTT, *fecE*, +756 | This study |
| CJ267 | CTCTAGCGACTCCTGATCTGATCC, *amiA*, +767 | This study |
| CJ268 | TGAATTGGTTGAAAGTATTCTGTGT, *amiA*, -1302 | This study |
| fatC1 | TAAAAGCAAACATACCAAGC; fatC; -9 | [3] |
| fatC2 | TAAAGAATAAGAAGCCACCC; fatC; +909 | [3] |
| ^a^Trim^R^, trimethoprim resistance; Mtx^R^, methotrexate resistance; Ery^R^, erythromycin resistance; | | |
| Kan^R^, kanamycin resistance; Sm^R^, streptomycin resistance; Spc^R^, spectinomycin resistance; | |  |
| Cm^R^, chloramphenicol resistance | |  |
| ^b^Referred to as D39Δ*codY* in original paper but rebaptized as TD75 in our laboratory due to new genotype information | | |
| ^c^*socY* refers to the presence of the *amiC^G1459T^* and *fatC^C496T^* mutations characterized previously [3] | | |
| ^d^*ami^CYbs0^* indicates mutation of the CodY binding site in front of the *ami* promoter, resulting in derepression  of *ami* in *codY*^+^ cells | | |
| ^e^Position is given with respect to the ATG of the corresponding gene; - and + indicate upstream and | | |
| downstream respectively | |  |
| ^C^ and ^A^ indicate, respectively, the co-transcribed and the reverse orientation of an inserted minitransposon | | |
| antibiotic resistance cassette with respect to the targeted gene | |  |

References

1. Mortier-Barrière I, de Saizieu A, Claverys JP, Martin B (1998) Competence-specific induction of *recA* is required for full recombination proficiency during transformation in *Streptococcus pneumoniae*. Mol Microbiol 27: 159-170.

2. Lefèvre JC, Claverys JP, Sicard AM (1979) Donor deoxyribonucleic acid length and marker effect in pneumococcal transformation. J Bacteriol 138: 80-86.

3. Caymaris S, Bootsma HJ, Martin B, Hermans PWM, Prudhomme M et al. (2010) The global nutritional regulator CodY is an essential protein in the human pathogen *Streptococcus pneumoniae*. Mol Microbiol 78: 344-360.

4. Johnston C, Martin B, Granadel C, Polard P, Claverys JP (2013) Programmed protection of foreign DNA from restriction allows pathogenicity island exchange during pneumococcal transformation. PLoS Pathogens 9: e1003178.

5. Hendriksen WT, Bootsma HJ, Estevão S, Hoogenboezem T, De Jong A et al. (2008) CodY of *Streptococcus pneumoniae*: link between nutritional gene regulation and virulence. J Bacteriol 190: 590-601.

6. Kloosterman TG, Hendriksen WT, Bijlsma JJ, Bootsma HJ, van Hijum SA et al. (2006) Regulation of glutamine and glutamate metabolism by GlnR and GlnA in *Streptococcus pneumoniae*. J Biol Chem 281: 25097-25109.
