# Supplementary material for: Co-Inactivation of GlnR and CodY Regulators Impacts Pneumococcal Cell Wall Physiology
Source: PLoS One. 2015 Apr 22;10(4):e0123702. doi: 10.1371/journal.pone.0123702 (PMC4406557; doi:10.1371/journal.pone.0123702)
Supplement: S2 Table — (DOCX) [file pone.0123702.s008.docx]

Table S2. Sensitivity to ampicillin and ceftriaxone of *S. pneumoniae* strain D39 and its *glnR*, *codY* (*fecE*) derivative,

measured as size of inhibition zone (mm).

| Antibiotic | Genotype^a^ | Antibiotic concentration (μg mL^-1^) | | | | | |
| --- | --- | --- | --- | --- | --- | --- | --- |
|  |  | 4 | 1 | 0.25 | 0.062 | 0.016 | 0.0039 |
| Ampicillin | wt | 26 ± 1.2 | 14 ± 1.4 | 2 ± 0.4 | 0 | 0 | 0 |
|  | *glnR^-^ codY^-^* (*fecE^-^*) | 38 ± 2.5 | 27 ± 1.8 | 19 ± 1.2 | 13 ± 0.9 | 7 ± 1.1 | 0 |
| Ceftriaxone | wt | 25 ± 1.9 | 18 ± 1,3 | 6 ± 0.6 | 0 | 0 | 0 |
|  | *glnR^-^ codY^-^* (*fecE^-^*) | 41 ± 2.2 | 31 ± 1.8 | 20 ± 1.4 | 15 ± 1.2 | 6 ± 2,1 | 0 |

^a^ Strains used from top to bottom: TD249, TK108.
