# Supplementary material for: Co-Inactivation of GlnR and CodY Regulators Impacts Pneumococcal Cell Wall Physiology
Source: PLoS One. 2015 Apr 22;10(4):e0123702. doi: 10.1371/journal.pone.0123702 (PMC4406557; doi:10.1371/journal.pone.0123702)
Supplement: S3 Table — (DOCX) [file pone.0123702.s009.docx]

Table S3 Peptidoglycan composition of wildtype and mutant strains.

| Strains^a^ | Peptidoglycan features (%) | | | | |
| --- | --- | --- | --- | --- | --- |
|  | wt | *fecE^-^ glnR^-^* | *glnR^-^ codY^-^ fecE^-^* | *fecE^-^ glnR^-^ codY^-^ amiC^-^* | *codY^-^ socY* |
| Monomers | 40.3 ± 2.9 | 39.7 ± 2.2 | 41.7 ± 1.1 | 42.5 ± 2.0 | 44.4 ± 1.5 |
| Dimers | 51.1 ± 0.3 | 52.6 ± 1.6 | 52.7 ± 0.6 | 49.0 ± 1.1 | 46.8 ± 1.5 |
| Trimers | 9.4 ± 3.6 | 9.0 ± 0.3 | 5.6 ± 0.4 | 8.7 ± 0.9 | 8.8 ± 3.0 |
| Degree of cross-linkage | 31.8 ± 2.2 | 32.3 ± 0.6 | 30.1 ± 0.6 | 30.3 ± 1.1 | 29.3 ± 1.2 |
| Unamidated peptides | 2.9 ± 0.5 | 2.3 ± 0.1 | 3.3 ± 0.4 | 2.1 ± 0.7 | 2.5 ± 0.1 |

^a^ Strains used from left to right: TD249, TD227, TK108, TD247 and TD75.
