# Supplementary material for: Co-Inactivation of GlnR and CodY Regulators Impacts Pneumococcal Cell Wall Physiology
Source: PLoS One. 2015 Apr 22;10(4):e0123702. doi: 10.1371/journal.pone.0123702 (PMC4406557; doi:10.1371/journal.pone.0123702)
Supplement: S1 Text — (DOCX) [file pone.0123702.s010.docx]

**Supporting Discussion (Text S1)**

**Exploring *codY* mutant cell morphology by microscopy**

*glnR codY* (*fecE*) mutant cells appeared to form short chains (Figure S2A; see also Figure S3G). Generally, the dimensions of mutant cells were larger than wildtype (length, width, area and volume; Figure S2B-E), with a greater spread of values. A large proportion of mutant cells formed chains, with the majority being over 4 cells in length, compared to wildtype cells which were all either mono- or diplococci (Figure S2G). The *codY^-^ socY* mutant also formed short chains, and showed perturbations in cell dimensions (Figure S2). Conversely, a *glnR fecE* mutant displayed characteristics similar to wildtype, as did a CEP_M_-*codY*^+^ complemented *glnR codY* (*fecE*) mutant (Figure S3).

**Previous documented examples of cell-wall alterations affecting pneumococcal sensitivity to PG-targeting agents**

A number of cell-wall alterations have been reported to alter pneumococcal sensitivity to certain agents targeting PG. Firstly, modifications of the alternating *N*-acetylmuramic acid (MurNAc) and *N*-acetylglucosamine (GlcNAc) residues that make the glycan chains in pneumococcal PG contribute to the well-known resistance of pneumococci to lysozyme, an important bacteriolytic enzyme of the innate immune system. These modifications include various degrees of deacetylation of the GlcNAc residues (by PgdA [1]) and *O*-acetylation of MurNAc residues (by Adr [2]) (Figure 5A, numbers 2 and 3 respectively). The latter is important for resistance to β–lactam antibiotics [2]. Secondly, formation of PG branches, which requires the MurM and MurN enzymes (Figure 5A, number 4), is crucial for β-lactam resistance. *murMN* mutant cells were thus shown to lack branched muropeptides and have reduced resistance to β-lactams [3] and other inhibitors of cell wall synthesis [4]. Thirdly, pneumococcal cells treated with the β–lactam clavulanate were shown to have increased sensitivity to DOC, lysozyme and penicillin. Analysis of PG from these cells revealed major alterations in stem peptide composition, including accumulation of D-alanyl-D-alanine residues, suggested to be as a consequence of suppressed DD-carboxypeptidase activity [5]. Fourthly, depletion of the penicillin-binding protein PBP2b, which plays a crucial role in PG synthesis, was reported to increase muropeptide branching and to result in long chains of oddly shaped cells. These cells were hypersensitive to exogenous LytA, while LytB was unable to separate daughter cells, accounting for the chaining phenotype observed [6]. Fifthly, it was recently shown that amidation of the second residue of the stem peptide, which alters the D-*iso*-Glu to D-*iso*-Gln (Figure 5A, number 5), was required for efficient PG cross-linking via transpeptidase activity [7,8]. This amidation is dependent on the amidotransferase enzyme MurT/GatD using L-Gln as donor (Figure 5B) [8-10].

The *glnR codY* (*fecE*) mutant shared a phenotype of sensitivity to lysozyme and β-lactams with cells depleted for MurT/GatD leading us to speculate on the possible alteration in stem peptide synthesis as a cause for the phenotype of the *glnR codY* (*fecE*) mutant. How could inactivation of *glnR* and *codY* alter stem peptide synthesis? Interestingly, GlnR and CodY share a regulatory target, the E dehydrogenase gene *gdhA* [11] which is involved in Glu/Gln metabolism, converting 2-oxoglutarate to Glu and vice-versa (Figure 5B). In addition, GlnR represses the Gln synthase gene *glnA*, which can synthesize Gln from Glu, and Glu from Gln and 2-oxoglutarate [11] (Figure 5B). Thus in the *glnR codY* (*fecE*) mutant, both of these genes are fully derepressed, potentially altering Glu/Gln metabolism and thus cellular pools of Glu/Gln. In stark contrast to the translocation of WTA across the cell membrane by the flippase TacF, which can only translocate fully mature, Cho-decorated WTA [12], published results demonstrate that non-amidated stem peptide precursors can be exported by FtsW/RodA. Thus, depletion of *murT/gatD* results in reduced amidation of the second stem peptide residue [8]. We therefore envisioned the possibility that imbalance of Gln/Glu pools resulting from derepression of crucial enzymes involved in Gln/Glu metabolism specifically interfered with amidation of the 2^nd^ stem peptide residue as Gln is necessary as a donor of an amide group for MurT/GatD-catalyzed reaction. However, HPLC analysis of muropeptide composition (Table S2 and Figure S5) provided no support to this hypothesis.

**References**

1. Vollmer W, Tomasz A (2000) The *pgdA* gene encodes for a peptidoglycan *N*-acetylglucosamine deacetylase in *Streptococcus pneumoniae*. J Biol Chem 275: 20496-20501.

2. Crisostomo MI, Vollmer W, Kharat AS, Inhulsen S, Gehre F et al. (2006) Attenuation of penicillin resistance in a peptidoglycan *O*-acetyl transferase mutant of *Streptococcus pneumoniae*. Mol Microbiol 61: 1497-1509.

3. Filipe SR, Tomasz A (2000) Inhibition of the expression of penicillin resistance in *Streptococcus pneumoniae* by inactivation of cell wall muropeptide branching genes. Proc Natl Acad Sci U S A 97: 4891-4896.

4. Filipe SR, Severina E, Tomasz A (2002) The *murMN* operon: a functional link between antibiotic resistance and antibiotic tolerance in *Streptococcuspneumoniae*. Proc Natl Acad Sci U S A 99: 1550-1555.

5. Severin A, Severina E, Tomasz A (1997) Abnormal physiological properties and altered cell wall composition in *Streptococcus pneumoniae* grown in the presence of clavulanic acid. Antimicrob Agents Chemother 41: 504-510.

6. Berg KH, Stamsas GA, Straume D, Havarstein LS (2013) Effects of low PBP2b levels on cell morphology and peptidoglycan composition in *Streptococcus pneumoniae* R6. J Bacteriol 195: 4342-4354.

7. Bui NK, Eberhardt A, Vollmer D, Kern T, Bougault C et al. (2012) Isolation and analysis of cell wall components from *Streptococcus pneumoniae*. Anal Biochem 421: 657-666.

8. Zapun A, Philippe J, Abrahams KA, Signor L, Roper DI et al. (2013) In vitro Reconstitution of Peptidoglycan Assembly from the Gram-Positive Pathogen *Streptococcus pneumoniae*. ACS Chem Biol 8: 2688-2696.

9. Figueiredo TA, Sobral RG, Ludovice AM, Almeida JM, Bui NK et al. (2012) Identification of genetic determinants and enzymes involved with the amidation of glutamic acid residues in the peptidoglycan of *Staphylococcus aureus*. PLoS Pathog 8: e1002508.

10. Munch D, Roemer T, Lee SH, Engeser M, Sahl HG et al. (2012) Identification and in vitro analysis of the GatD/MurT enzyme-complex catalyzing lipid II amidation in *Staphylococcus aureus*. PLoS Pathog 8: e1002509.

11. Kloosterman TG, Hendriksen WT, Bijlsma JJ, Bootsma HJ, van Hijum SA et al. (2006) Regulation of glutamine and glutamate metabolism by GlnR and GlnA in *Streptococcus pneumoniae*. J Biol Chem 281: 25097-25109.

12. Damjanovic M, Kharat A, Tomasz A, Vollmer W (2007) The essential *tacF* gene is responsible for the choline-dependent growth phenotype of *Streptococcus pneumoniae*. J Bacteriol 189: 7105-7111.
